# CD32-Expressing CD4 T Cells Are Phenotypically Diverse and Can Contain Proviral HIV DNA

**DOI:** 10.3389/fimmu.2018.00928

**Published:** 2018-05-04

**Authors:** Genevieve E. Martin, Matthew Pace, John P. Thornhill, Chansavath Phetsouphanh, Jodi Meyerowitz, Morgane Gossez, Helen Brown, Natalia Olejniczak, Julianne Lwanga, Gita Ramjee, Pontiano Kaleebu, Kholoud Porter, Christian B. Willberg, Paul Klenerman, Nneka Nwokolo, Julie Fox, Sarah Fidler, John Frater

**Affiliations:** ^1^Peter Medawar Building for Pathogen Research, Nuffield Department of Medicine, University of Oxford, Oxford, United Kingdom; ^2^Division of Medicine, Wright Fleming Institute, Imperial College, London, United Kingdom; ^3^Department of Genitourinary Medicine and Infectious Disease, Guy’s and St Thomas’ NHS Foundation Trust, London, United Kingdom; ^4^HIV Prevention Research Unit, South African Medical Research Council, Durban, South Africa; ^5^Uganda Virus Research Institute (MRC), Entebbe, Uganda; ^6^Research Department of Infection and Population Health, University College London, London, United Kingdom; ^7^NIHR Biomedical Research Centre, University of Oxford, Oxford, United Kingdom; ^8^Chelsea and Westminster Hospital, London, United Kingdom

**Keywords:** HIV, reservoir, CD32, primary HIV infection, HIV DNA

## Abstract

Efforts to both characterize and eradicate the HIV reservoir have been limited by the rarity of latently infected cells and the absence of a specific denoting biomarker. CD32a (FcγRIIa) has been proposed to be a marker for an enriched CD4 T cell HIV reservoir, but this finding remains controversial. Here, we explore the expression of CD32 on CD3^+^CD4^+^ cells in participants from two primary HIV infection studies and identify at least three distinct phenotypes (CD32^low^, CD32^+^CD14^+^, and CD32^high^). Of note, CD4 negative enrichment kits remove the majority of CD4^+^CD32^+^ T cells, potentially skewing subsequent analyses if used. CD32^high^ CD4 T cells had higher levels of HLA-DR and HIV co-receptor expression than other subsets, compatible with their being more susceptible to infection. Surprisingly, they also expressed high levels of CD20, TCRαβ, IgD, and IgM (but not IgG), markers for both T cells and naïve B cells. Compared with other populations, CD32^low^ cells had a more differentiated memory phenotype and high levels of immune checkpoint receptors, programmed death receptor-1 (PD-1), Tim-3, and TIGIT. Within all three CD3^+^CD4^+^CD32^+^ phenotypes, cells could be identified in infected participants, which contained HIV DNA. CD32 expression on CD4 T cells did not correlate with HIV DNA or cell-associated HIV RNA (both surrogate measures of overall reservoir size) or predict time to rebound viremia following treatment interruption, suggesting that it is not a dominant biomarker for HIV persistence. Our data suggest that while CD32^+^ T cells can be infected with HIV, CD32 is not a specific marker of the reservoir although it might identify a population of HIV enriched cells in certain situations.

## Introduction

A cure for HIV infection is contingent on targeting and neutralizing latently infected cells that persist despite effective antiretroviral therapy (ART). These cells are predominantly resting memory CD4 T cells containing transcriptionally silent, integrated proviral HIV DNA. Activation of these cells results in HIV production and disease progression in the absence of ART. Latently infected cells are extremely rare (0.0001–0.1% of CD4 T cells), complicating their study. Accordingly, there has been a research effort to identify cell surface markers that identify latently infected cells to facilitate their characterization and targeting. For example, immune checkpoint receptors (ICRs) (1) such as programmed death receptor-1 (PD-1), T cell immunoglobulin (Ig) and mucin domain-containing molecule (Tim)-3, and T cell immunoreceptor with Ig and ITIM domains (TIGIT) are associated with an exhausted effector state (2–5), and their expression correlates with HIV disease progression and viral rebound following treatment interruption (TI) (4, 6, 7). ICRs have been reported to be highly expressed on cells comprising the HIV reservoir (8, 9), but are not discriminatory.

CD32a (FcγRIIa)—a low-affinity IgG receptor expressed on myeloid cells and granulocytes (10), has been proposed as a surface marker for latent HIV infection (11). While this finding is of extremely high interest, the expression of CD32 on CD4 T cells remains controversial, and a role for low-affinity Fc receptors on CD4 T cells has not been well-established (12–14). Although CD4 T cells are generally not considered to express CD32, expression may be induced by activation *in vitro* (15, 16). Expression of CD32b on memory CD8 T cells in murine infection models is associated with reduced cytotoxicity and expansion, reversible on CD32b blockade (17). This has interesting parallels with other co-inhibitory pathways, raising the possibility that CD32 isoforms may have a similar regulatory role on activated, antigen experienced CD4 T cells.

Here, we investigate CD32 expression on CD3^+^CD4^+^ cells and identify distinct populations that express this marker. We also characterize CD32-expressing CD4 T cells in the blood of individuals treated during primary HIV infection (PHI) [a group of interest due to an association with post treatment virological remission and a more labile reservoir (18–20)] and explore associations with overall reservoir size, cell phenotype, and clinical progression.

## Materials and Methods

### Participants

Participants with PHI were recruited as part of the HEATHER (HIV Reservoir targeting with Early Antiretroviral Therapy) cohort. PHI was identified through one of the following criteria: (a) HIV-1 positive antibody test within 6 months of a HIV-1 negative antibody test, (b) HIV-1 antibody negative with positive PCR (or positive p24 Ag or viral load detectable), (c) RITA (recent incident assay test algorithm) assay result consistent with recent infection, (d) equivocal HIV-1 antibody test supported by a repeat test within 2 weeks showing a rising optical density, and (e) having clinical manifestations of symptomatic HIV seroconversion illness supported by antigen positivity. For inclusion in the cohort, participants with identified PHI commenced ART within 3 months of diagnosis and did not have co-infection with hepatitis B or C. Date of seroconversion was estimated as the midpoint of the dates of the most recent negative or equivocal test and positive test (criteria a and d above), the date of the test (b and e) or 120 days before the test date (c, the recency period of this assay). For our study, cryopreserved peripheral blood mononuclear cells (PBMCs) were used from the closest pre-therapy sample to seroconversion (baseline) and from a sample 9–15 months after commencement of ART (1 year).

Time to rebound analyses was conducted with a subset of participants from the SPARTAC (Short Pulse Antiretroviral Therapy at HIV Seroconversion) trial (EudraCT Number: 2004-000446-20). This was a multicenter, randomized controlled trial of short course ART during PHI, the full design of which is described elsewhere (21). The criteria used to define PHI in this trial are similar to above. In brief, this trial enrolled adults with PHI from 25 sites in Australia, Brazil, Ireland, Italy, South Africa, Spain, Uganda, and the UK. Participants with PHI were randomized to receive no immediate ART (standard of care), 12 or 48 weeks of ART, after which they underwent a TI. The primary trial endpoint was a composite of CD4 T cell count less than 350 cells/μL or the initiation of long-term ART for any reason. Cryopreserved PBMCs were used from participants who received 48 weeks of ART and were virologically suppressed to <400 copies/mL at the time of TI. Participants were included based on sample availability at the time of TI, and date of seroconversion was estimated as calculated previously (7).

### Flow Cytometry and Cell Sorting

Cryopreserved PBMCs were thawed in RPMI-1640 medium supplemented with 10% FBS, l-glutamine, penicillin, and streptomycin (R10) containing 2.7 Kunitz units/mL of DNAse (Qiagen). For analysis of memory phenotype and ICR expression, cells were stained in BD Horizon Brilliant Stain Buffer (BD) containing all antibodies and Live/Dead Near IR at 1 in 300 dilution (Life Technologies) at 4°C for 30 min. PBMCs were stained with the following antibodies: CD32 PE-Cy7 (FUN-2), CD3 Brilliant Violet (BV) 570 (UCHT1), CCR7 Pacific Blue (G043H7), CD27 AlexaFluor700 (M-5271) (all BioLegend), CD4 BV605 (RPA-T4), CD8 BV650 (RPA-T8) (BD), PD-1 PE-eFluor 610 (eBioJ105), CD45RA FITC (HI100), TIGIT PerCP-eFluor710 (MBSA43) (all eBioscience), and Tim-3 PE (344823) (R&D). Isotype controls for CD32 were prepared using an irrelevant IgG2bκ antibody (MPC-11) (BioLegend). For time to rebound analyses, cells were stained as above in PBS with 5% FBS and 1 mM EDTA (FACS buffer) containing Live/Dead Near IR, CD32 PE-Cy7, PD-1 PE-eFluor610, TIGIT PerCP-eFluor710 as well as the following antibodies: CD3 FITC (UCHT1) (BioLegend) and CD4 eFluor450 (OKT4) (eBioscience). For measurement of HLA-DR expression, cells were stained for 20 min at room temperature with LiveDead Near IR and the following antibodies: CD3 BV570, CD4 BV605, CD8 BV650, CD32 PE (FUN-2) (BioLegend) and HLA-DR AlexaFluor700 (MAB) (BD). Samples for HIV co-receptor stains were stained with LiveDead Near IR, CD32 PE-Cy7, CD3 FITC, and CD4 eFluor450. Following this, cells were incubated at 37°C for 20 min with anti-CCR5 PE (C57BL/6) (BD) or at 4°C for 30 min with anti-CXCR4 APC (12G5) (Miltenyi).

For measurement of lineage marker expression on CD32-expressing CD4 T cells, PBMCs were stained as above in R10 with antibodies as listed in Panels 1–6 below. A CD32 isotype control was used as above, and gates for lineage markers were determined using a fluorescence minus one or the positive population from bulk PBMC.

Panel 1—CD11b, CD16, CD19, CD20, CD123, and TCRγδ staining. PBMCs were stained with LiveDead Near IR, CD3 AlexaFluor700 (UCHT1), CD4 APC (RPA-T4) (both BioLegend), CD32 PE-Cy7, CD14 VioBlue (TUK4) (Miltenyi), either CD11b FITC (M1/70.15.11.5) (Miltenyi), CD16 FITC (CB16) (eBioscience), CD19 FITC (LT19) (Miltenyi), CD20 FITC (2H7) (BioLegend), CD123 PE (9F5) (BD Bioscience) or TCRγδ PE (11F2) (Miltenyi).
Panel 2—CD8, CD15, CD56 staining. PBMCs were stained with LiveDead Near IR, CD3 AlexaFluor700, CD4 APC, CD32 PE-Cy7, and CD14 FITC (HCD14) (BioLegend) and CD8 eFluor450 (OKT8), CD15 Pacific Blue (W6D3), or CD56 BV421 (HCD56) (all BioLegend).
Panel 3—CD235a staining. PBMCs were stained with Fixable Viability Dye eFluor 506 (eBioscience), CD3 FITC, CD4 APC, CD32 PE-Cy7, CD14 VioBlue, and CD235a APC-Cy7 (HI264) (BioLegend).
Panel 4—CD36 staining. PBMCs were stained with LiveDead Near IR, CD3 FITC, CD32 PE-Cy7, CD4 AlexaFluor700 (SK3) (BioLegend), CD14 VioBlue, and CD36 APC (AC106) (Miltenyi).
Panel 5—TCR αβ staining. PBMCs were stained with LiveDead Near IR, CD3 FITC, CD4 APC, CD32 PE, and CD14 VioBlue. Cells were then permeabilized using BD FACS Permeabilizing solution 2 as per the manufacturer’s protocol before washing and staining with TCR αβ PE-Cy7 (IP26) (BioLegend).
Panel 6—Ig staining. PBMCs were stained with LiveDead Near IR, CD3 AlexaFluor700, CD4 APC, CD32 PE-Cy7, CD14 VioBlue, CD20 FITC and IgD PE (IA6-2), IgG PE (HP6017), or IgM PE (MHM-88) (all BioLegend).

Phenotyping of fresh lymphocytes was performed within 3 h of blood collection using 1 mL of ACD anticoagulated blood. Erythrocytes were lysed at 4°C for 10 min in 10 mL of ammonium-chloride-potassium (ACK) buffer (Gibco) and washed before staining in FACS buffer as above using Panel 1.

All samples were acquired on an LSR II (BD). The same machine was used for all experiments with daily calibration with Cytometer Setup and Tracking beads (BD) to maximize comparability between days. Rainbow Calibration Particles (BioLegend) were also used for cohort phenotyping to minimize batch-to-batch variability. Data were analyzed using FlowJo Version 10.8.0r1 (Treestar). In some cases for the CD32^high^ populations, cell numbers were very low. To ensure accuracy in the phenotyping of this population, any populations where there were five or fewer events were excluded from further phenotyping analyses. (Of note, the overall phenotype of this population did not change if this threshold was changed to fewer than 25 or 50 events.)

For sorting experiments, thawed PBMCs or tonsillar cells were stained in R10 at 4°C using Live/Dead Near IR, CD32 PE-Cy7, CD3 FITC and either (a) CD4 APC and CD14 VioBlue or (b) CD4 eFluor450. Cells were sorted using a Mo-Flo XDP into four CD3^+^CD4^+^ populations: CD32^−^, CD32^low^, CD32^+^CD14^+^, and CD32^high^ cells.

### CD4 Isolation Using EasySep Kit

Peripheral blood mononuclear cells were thawed as above. For Fc blocking experiments, this was split into two aliquots. One aliquot was treated for 10 min at room temperature with Human TruStain FcX (BioLegend) according to the manufacturer’s protocol while the other was left untreated. A fraction of each aliquot was removed for CD32 staining. The remainder was used for CD4 enrichment *via* negative selection with the EasySep Human CD4 Enrichment Kit (StemCell) per the manufacturer’s protocol. PBMCs or enriched CD4s were stained as above with Live/Dead Near IR, CD32 PE-Cy7, CD3 FITC, and CD4 eFluor450.

### Measurement of HIV DNA

For measurement of HIV DNA in bulk CD4 T cells, CD4 T cells were enriched from cryopreserved PBMCs as above or using Dynabeads Untouched Human CD4 T Cell Enrichment kit (Invitrogen). DNA was extracted from PBMCs or enriched CD4 T cells (using QIAamp Blood Mini Kit, Qiagen) and sorted CD4 T cell subsets (using QIAamp DNA Micro Kit or Mini Kit, both Qiagen) for use as input for qPCR assays. Copies of HIV-1 DNA were quantified and normalized to number of input cells (as determined by albumin PCR), by a previously described assay (22, 23) with both qPCR assays performed in triplicate. For sorted populations, PCR reactions for both albumin and total HIV were performed in triplicate for the CD32^−^ population and in duplicate for the remaining populations, except where otherwise noted. Negative sample wells were replaced with zeros when averaging replicate values.

### Unspliced HIV-RNA Transcript Quantitation

RNA was isolated using the Qiagen AllPrep DNA/RNA Mini kit as per the manufacturer’s recommendations with the addition of two on-column double DNase digestions. The RNA assay performed as previously described (6). Briefly, complementary DNA was subjected to two rounds of PCR using semi-nested primers. The first reaction was a 15-cycle endpoint reaction using MH535 (5′-AACTAGGGAACCCACTGCTTAAG-3′) and SL20 (5′-TCTCCTTCTAGCCTCCGCTAGTC-3′) primers at a concentration of 400 nM each. The second reaction was a 40-cycle quantitative reaction (forward primer 5′-TAAAGCTTGCCTTGAGTGCT, SL20 and the probe FAM—5′-AGTRGTGTGTGCCCGTCTGTTG-3′—BHQ-1; 330 nM each), performed using a Roche Lightcycler 480. The HIV standard was generated by *in vitro* transcribing the plasmid Sp5-NL4.3 (generously provided by the lab of D. Purcell) using the RiboMax Large Scale SP6 RNA production System (Promega). HIV RNA standards were diluted in 10 ng/µL uninfected PBMC RNA. Any background from wells without reverse transcriptase was subtracted from final measurements. HIV RNA copies were normalized to RNA weight measured *via* Nanodrop.

### Statistical Analysis

Continuous variables were compared between groups using non-parametric tests throughout. Where three groups were compared, a Kruskal–Wallis test was used; pairwise comparisons were performed on all combinations of groups only if the overall test *p*-value was <0.05. Correlative analyses were performed using Spearman’s rank correlation. Time to viral rebound was assessed as time from TI to the first of two consecutive VL measurements >400 copies/mL (the limit of detection of the assay used at some trial sites). Individuals who did not rebound were censored at the date of the last VL measurement. Time to viral rebound was visualized with Kaplan–Meier curves stratified at the median and associations examined using Cox proportional hazard models. Analyses were performed using GraphPad Prism (GraphPad Software, La Jolla, CA, USA) version 6.0f or R version 3.2.2.

## Results and Discussion

### Depletion of CD32-Expressing CD4 T Cells by Negative Bead Selection due to the Expression of Non-T Cell Markers

**Figure 1:**
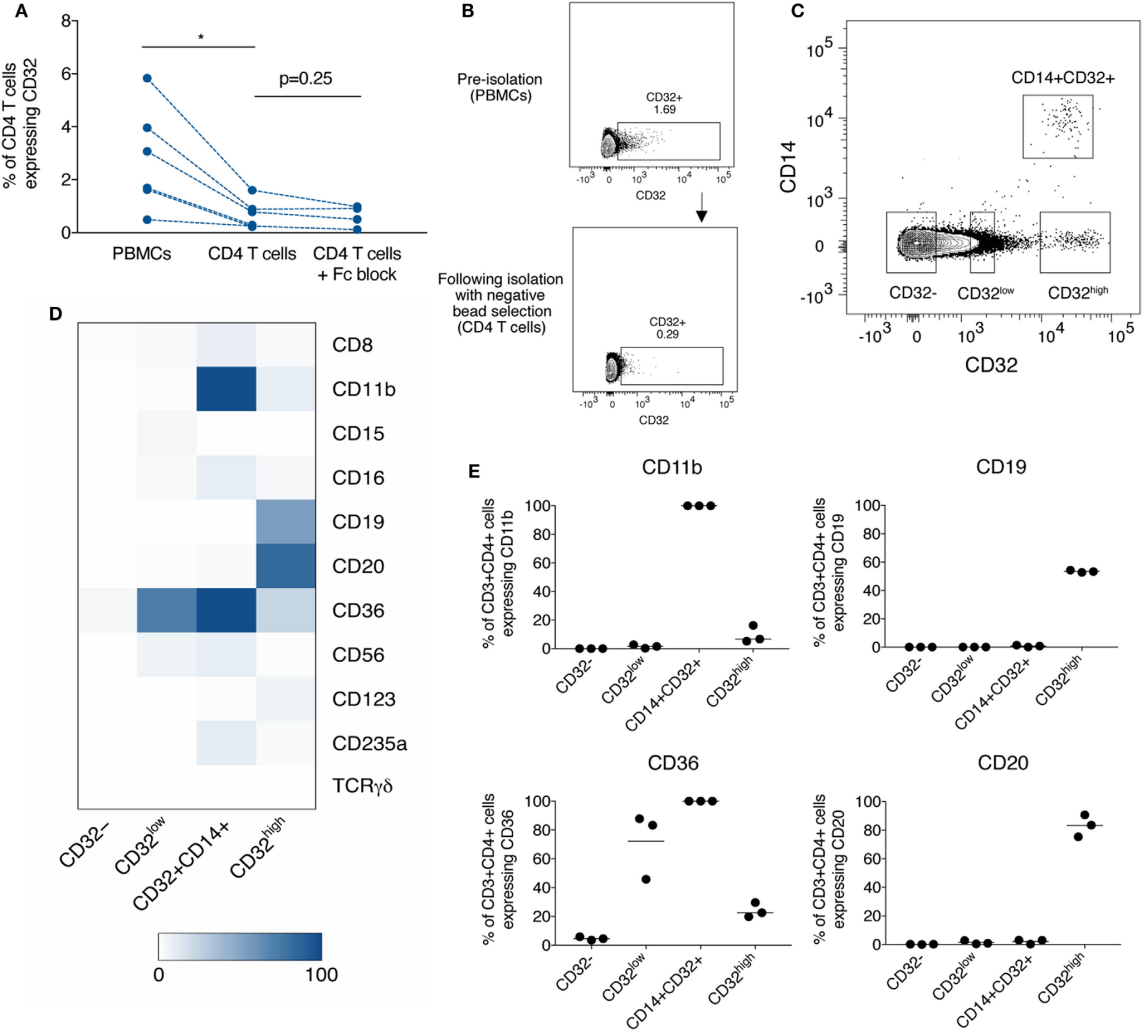
Depletion of diverse populations of CD32-expressing CD4 T cells by negative bead selection due to the expression of non-T cell markers. **(A)** The frequency of CD32^+^CD3^+^CD4^+^ T cells when measured in peripheral blood mononuclear cells (PBMCs), or in CD4 T cells purified by negative bead selection, with and without Fc block prior. **(B)** Representative gating of data shown in panel **(A)**. **(C)** Gating of three CD32-expressing populations based on differing levels of expression and CD14: CD32^low^, CD32^+^CD14^+^, and CD32^high^. **(D)** Heat-map showing the percentage expression of markers used in CD4 negative bead isolation kits on subsets of CD3^+^CD4^+^ T cells [defined in panel **(C)**]. Expression was measured by flow cytometry, and values are shown as the mean of three individual donors. **(E)** Percentage expression of CD11b, CD19, CD36, and CD20 on CD3^+^CD4^+^ subpopulations as shown in panel **(D)**. Bar is shown at the mean.

The experimental study of CD4 T cells frequently incorporates a commercial bead-based negative selection kit in the laboratory protocol to produce a purified population of CD4 T cells from bulk PBMCs. We identified that CD32 expression on purified CD4 T cells isolated by negative bead selection was consistently lower than in CD4 T cells in PBMCs. To test if the cell purification kits depleted CD32-expressing CD4 T cells, we compared frequencies of CD32^+^CD4 T cells before and after negative selection by flow cytometry. We found a significant decrease in CD32 expression after negative selection (Figures 1A,B), which was not prevented by pre-treatment with an Fc blocking reagent to control for non-specific antibody binding (Figure 1A).

As these data suggested that CD32^+^CD4 T cells express other surface molecules specifically depleted by the negative selection kit (which does not contain anti-CD32 antibodies), we looked for co-expression with CD32 of a panel of lineage-defining markers used in CD4 T cell selection kits from several manufacturers. These were as follows: CD8, CD11b, CD14, CD15, CD16, CD19, CD20, CD36, CD56, CD123, TCRγδ, and CD235a.

The monocyte marker, CD14, distinguished a separate population of CD32^+^CD14^+^ cells. The remaining CD14^−^CD32^+^ cells could be divided into two distinct populations, CD32^low^ and CD32^high^ (Figure 1C). We therefore further characterized lineage marker expression in these four populations—CD32^−^, CD32^low^, CD32^high^, and CD32^+^CD14^+^ and found clear phenotypic differences, consistent with four distinct cell populations (Figure 1D; Figure S1 in Supplementary Material). CD32^−^CD4 T cells did not express significant levels of any tested lineage markers. CD32^+^CD14^+^ cells all expressed CD11b (Figure 1E) and were found almost entirely within a monocyte-rich FSC/SSC region (Figure S2 in Supplementary Material), consistent with these cells being monocytes despite high levels of CD3 expression. It is possible that these cells are monocytes which have acquired CD3 *via* trogocytosis. The CD32^low^ population expressed high levels of CD36 (Figure 1E), a scavenger receptor typically found on platelets and monocytes in blood but which has also been reported on CD4 T cells interacting with platelets (24). The CD32^high^ cells expressed high levels of CD19 and CD20, traditional markers of B cells (Figure 1E; representative staining in Figures S1B,C in Supplementary Material).

**Figure 2:**
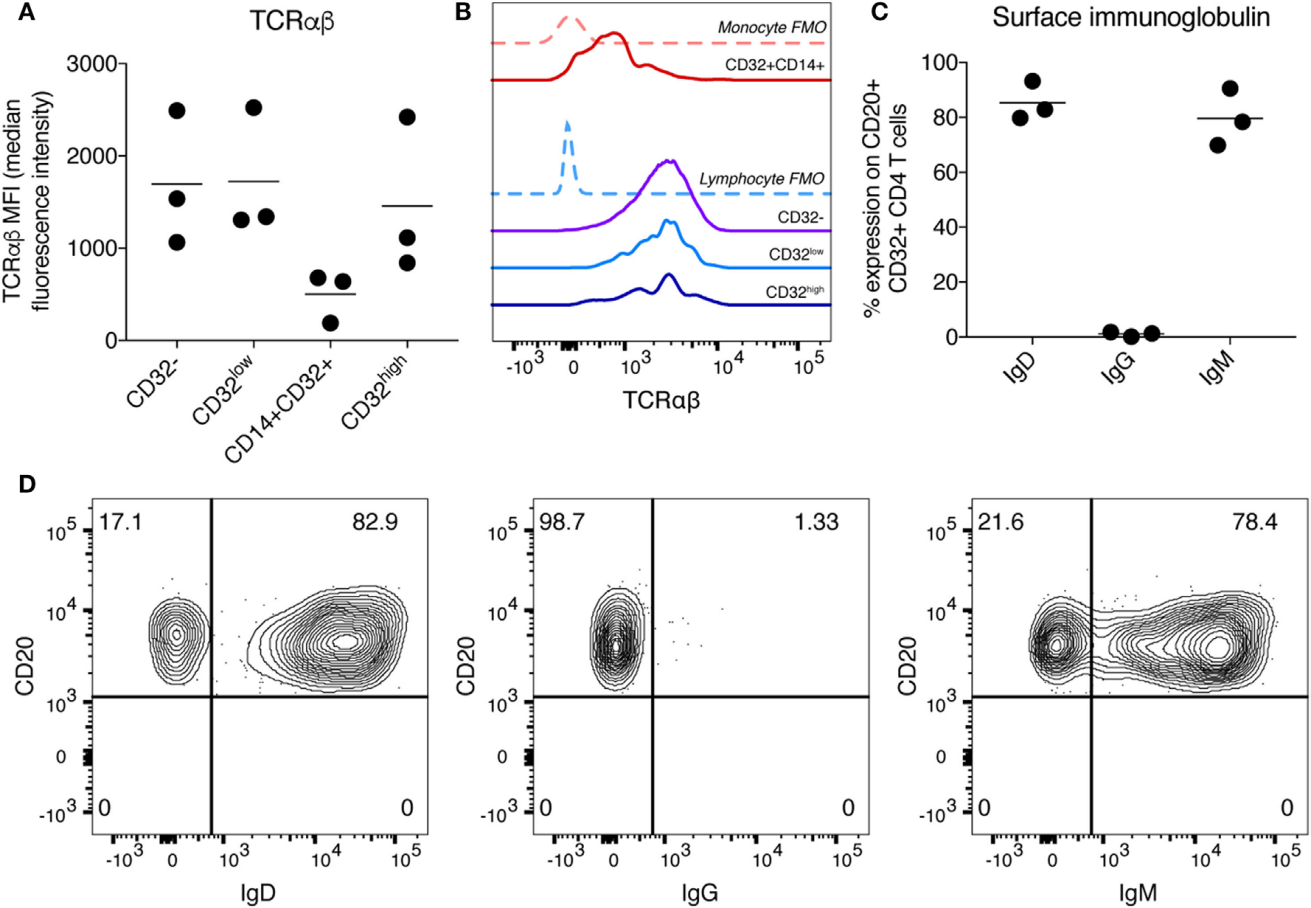
Characterization of T cell receptor and immunoglobulin (Ig) expression on CD32^high^CD3^+^CD4^+^ T cells. **(A)** Expression of TCRαβ on CD32-expressing CD3^+^CD4^+^ T cell populations was measured by flow cytometry and is shown here as median fluorescence intensity. Bar is shown at the mean. **(B)** Histogram showing TCRαβ staining on CD32-expressing CD3^+^CD4^+^ T cell populations of one sample shown in panel **(A)**. Fluorescence minus one (FMO) controls are shown as dashed lines and individual populations as solid lines. **(C)** Percentage expression of Ig was measured on CD3^+^CD4^+^CD32^high^CD20^+^ cells. Expression of IgD, IgG, and IgM is shown. Expression was measured by flow cytometry, and bar is shown at the mean. **(D)** Representative staining of data shown in panel **(C)**.

We focused on further characterization of the CD32^high^ population, which has been reported to have the highest level of persistent HIV infection (11). As these cells also expressed CD3, CD4, CD19, and CD20, we were interested to see whether these cells expressed other markers associated with B or T cell lineage and function. To confirm that cells expressing CD3, CD4, and CD32 were T cells, we stained for TCRαβ expression. As we would expect for T cell populations, the CD32^−^, CD32^low^, and CD32^high^ populations all expressed similar levels of TCRαβ, while the CD32^+^CD14^+^ cells expressed very little, consistent with this subpopulation being monocytes (Figures 2A,B). We examined the expression of IgG, IgD, and IgM on the CD32^high^ cells. We found the CD32^high^ population, which co-express CD20 and TCRαβ, expressed high levels of IgD and IgM, but not IgG, similar to a naïve B cell phenotype (25) (Figures 2C,D). These results, surprisingly, show that the CD32^high^ population express both T and B cell phenotypic markers. While rare, CD20-expressing T cells populations have been described in healthy individuals and in a number of non-malignant disease states (26–31). Of note, the cells we described differ from these reports which generally expressed CD20 but not CD19.

Other investigators noting CD3 and CD20 co-expression have attempted to determine if this finding might be artefactual and secondary to contamination, cell doublets, cryopreservation or cell separation techniques (26, 31). Accordingly, for all experiments, cells were tightly gated on singlets (as shown in Figure S3A in Supplementary Material), to minimize the possibility of a doublet artefact in our data. Of note, performing a second doublet exclusion step does not change the frequency or phenotype of these cells (Figure S3B in Supplementary Material). To exclude the possibility that this phenotype was an artefact of density gradient separation or cryopreservation of PBMCs we stained fresh, whole blood from healthy donors and observed a similar pattern of CD32 and CD20 co-expression on CD3^+^CD4^+^ cells (Figure S4 in Supplementary Material).

Overall, these results have clear implications for those exploring the findings of Descours et al (11) as negative bead isolation of CD4 T cells will not only reduce the overall number of CD32^+^CD3^+^CD4^+^ cells but also affect the composition of the overall CD32-expressing population.

### CD32-Expressing CD3^+^CD4^+^ Cells Differ in Their Expression of ICRs and Memory Phenotype

**Figure 3:**
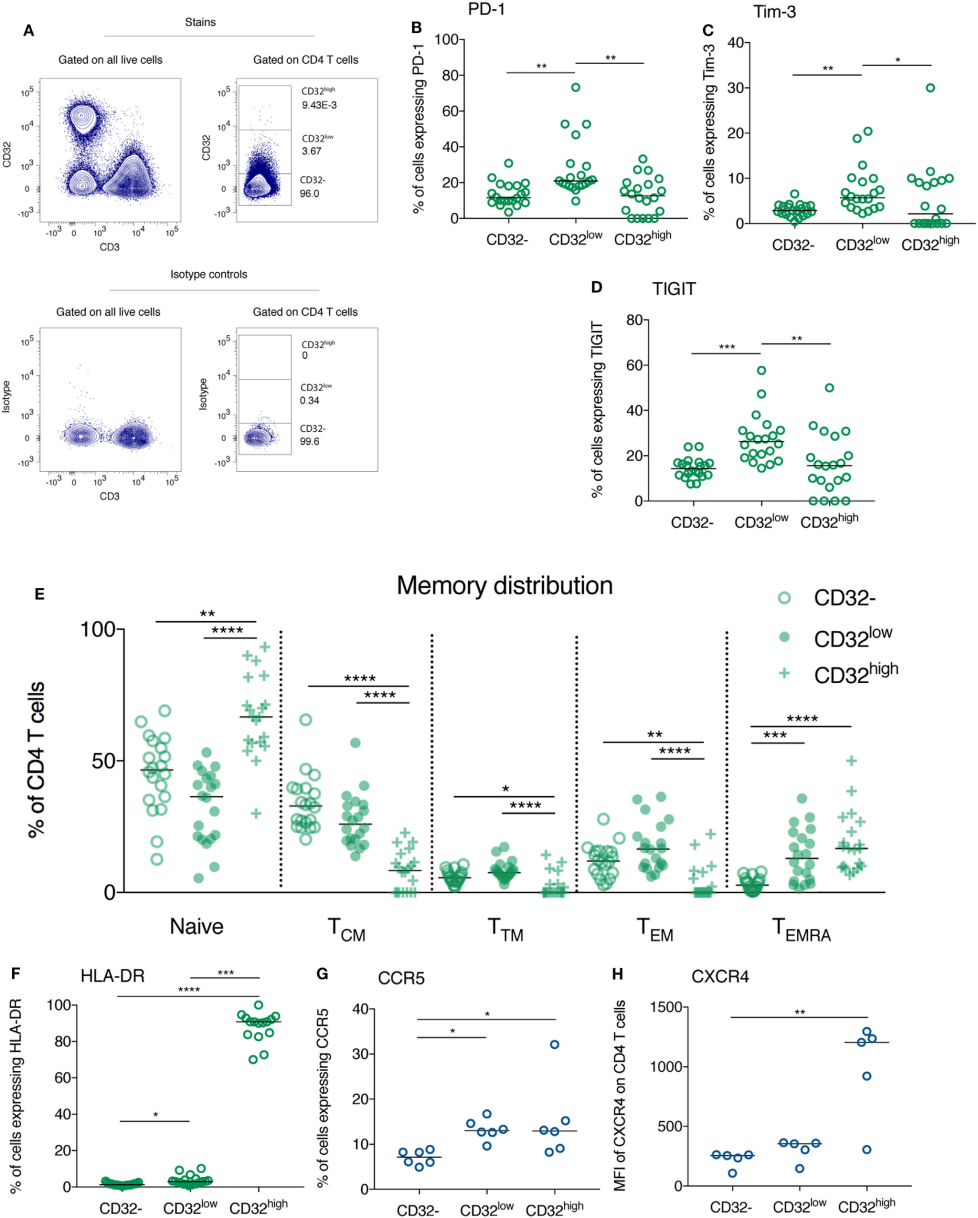
CD32-expressing CD3^+^CD4^+^ cells differ in their expression of immune checkpoint receptors, the activation marker HLA-DR and HIV co-receptors, as well as their memory phenotype. Representative gating of CD32-expressing populations on CD4 T cells from an HIV^+^ individual is shown (top panel) in panel **(A)**, relative to isotype control (bottom panel). CD32^−^, CD32^low^, and CD32^high^CD3^+^CD4^+^ T cell populations were compared with regards to their expression of programmed death receptor-1 (PD-1) **(B)**, Tim-3 **(C)**, TIGIT [**(D)**; all *n* = 20], and HLA-DR [**(F)**, *n* = 19]. For panel **(F)**, four samples were excluded from the analysis of the CD32^high^ population because there were five or fewer events. These same populations were compared with regards to their memory phenotype. The percentage of each population composed of naïve (CD45RA^+^CCR7^+^), central memory (T_CM_; CD45RA^−^CCR7^+^), transitional memory (T_TM_; CD45RA^−^CCR7^−^CD27^+^), effector memory (T_EM_; CD45RA^−^CCR7^−^CD27^−^), and T_EMRA_ cells (CD45RA^+^CCR7^−^) was quantified by flow cytometry and is shown in panel **(E)** (*n* = 20). The comparisons are all shown in HIV^+^ individuals at 1 year following the initiation of antiretroviral therapy. The expression of HIV co-entry receptors CCR5 [**(G)**; *n* = 6] and CXCR4 [**(H)**; *n* = 5] was compared between CD32^−^, CD32^low^, and CD32^high^CD3^+^CD4^+^ T cell populations in healthy individuals. Throughout, a Kruskal–Wallis test was used to compare all three groups; pairwise comparisons were performed on all combinations of groups only if the overall test *p*-value was < 0.05; ****p < 0.0001, ***p = 0.0001–0.001, **p = 0.001–0.01, *p = 0.01–0.05; for ease of interpretation if *p* ≥ 0.05 for any comparison this is not shown on these plots.

We further explored the phenotype of CD32-expressing CD4^+^ populations in individuals who commenced ART during PHI from the HEATHER (7) cohort. Key demographic and clinical characteristics are shown in Table 1. In these individuals, we studied both CD32^low^ and CD32^high^CD3^+^CD4^+^ populations separately without using cell enrichment kits (representative gating shown in Figure 3A) and imposing a conservative lymphocyte gate to eliminate the CD32^+^CD14^+^ population (Figure S2 in Supplementary Material, with doublet exclusion as in Figure S3C in Supplementary Material).

**Table 1:** Demographic and clinical characteristics of participants included in clinical studies.

	HEATHER (*n* = 20)^a^	SPARTAC (*n* = 19)^b^
**Sex**		
• Male	20 (100%)	5 (26%)
Age	33 (28–40.8)	27 (22–40)
**Country**		
• UK	20 (100%)	4 (21%)
• South Africa/Uganda	0	13 (68%)
• Other	0	2 (11%)
Time between estimated date of seroconversion and antiretroviral therapy (ART) initiation (days)	41.5 (22.5–55.3)	109 (76–124)
Time of sampling (weeks since ART initiation)	52.5 (52.0–57.6)	47.9 (47.6–48.0)
Baseline CD4 T cell count (cells/μL)	514 (376–628)	634 (535–764)
Baseline HIV RNA (log_10_ copies/mL)	6.32 (4.55–6.76)	4.32 (3.68–4.95)

*Demographic and clinical characteristics of participants included in clinical studies. Values given represent *n* (%) for categorical variables and median (interquartile range) for continuous variables*.

*^a^Data from these individuals contribute to Figures 3A–E and 5A,C and Figures S5A–D, S6A, S9, and S10 in Supplementary Material*.

*^b^Data from these individuals contribute to Figure 5 and Table S4 in Supplementary Material*.

The expression of the ICRs PD-1 and TIGIT has been reported to mark a population of CD3^+^CD4^+^ T cells enriched for HIV DNA, although the fold enrichment in HIV DNA is substantially lower than described for CD32 (8, 9, 11). We found that in treated HIV^+^ individuals, the expression of PD-1, Tim-3, and TIGIT was elevated on CD32^low^ CD3^+^CD4^+^ cells compared with their CD32^−^ and CD32^high^ counterparts (Figures 3B–D).

We compared the memory phenotype of CD32^−^, CD32^low^, and CD32^high^ CD3^+^CD4^+^ cell subsets during treated PHI. CD32^low^CD4 T cells had lower proportions of naïve and central memory (T_CM_) T cells than their CD32^−^ counterparts. By contrast, transitional memory (T_TM_), effector memory (T_EM_), and T_EMRA_ cells comprised a greater portion of the CD32^low^ CD4 T cell pool than for CD32^−^CD4 T cells (Figure 3E), reflecting a more differentiated memory phenotype. By contrast, the CD32^high^ population was largely comprised of cells which expressed CD45RA marking a naïve or T_EMRA_ phenotype. It is interesting that central memory cells—the memory subset with the highest level of proviral DNA (9, 32)—were most common among CD32^−^ cells and less frequent among both CD32^low^ and CD32^high^ cells. Furthermore, CD32^high^ cells contained lower levels of PD-1, Tim-3, and TIGIT than CD32^low^ cells suggesting that this may be a distinct population of HIV-infected cells than those enriched ICR expressing populations (8, 9, 11).

The overall phenotype of these cells did not differ between participant groups. This same pattern of ICR expression was observed before ART initiation (Figures S5A–C in Supplementary Material) and in healthy controls (Figures S5E–G in Supplementary Material), except for Tim-3 which was expressed at very low levels in CD4 T cells from controls. Similarly, these cells had the same memory phenotype before ART initiation (Figure S6A in Supplementary Material) and in healthy controls (Figure S6B in Supplementary Material). The distinct phenotype of CD32^−^, CD32^low^, and CD32^high^ populations with regards to memory differentiation and ICR expression adds to the lineage marker evidence that these are distinct cellular populations and are found in healthy as well as HIV-infected individuals.

### CD32^high^ CD3^+^CD4^+^ T Cells Are Activated and Express High Levels of HIV Co-Receptors

Among treated HIV-infected individuals (*n* = 19, Table S1 in Supplementary Material) CD32^high^ CD3^+^CD4^+^ cells expressed markedly elevated levels of the activation marker HLA-DR compared with both other populations (Figure 3F; also shown at baseline in Figure S5D in Supplementary Material). In healthy controls, the expression of CCR5 and CXCR4, the co-receptors used by HIV for cellular entry, was increased on CD32^high^CD4 T cells (Figures 3G,H). For CCR5, this was also observed on the CD32^low^ population.

The HIV reservoir is considered to be comprised of resting CD4 T cells (9, 33–35). The elevated expression of the activation marker HLA-DR on CD32-expressing CD4 T cells observed here is, therefore, potentially surprising. This finding is, however, consistent with two recent reports of elevated HLA-DR on bulk CD32^+^CD4 T cells from HIV-infected individuals (36, 37). Upregulation of CD32 expression on CD4 T cells with *in vitro* activation has been previously reported (15, 16), and it is possible that CD32 expression might mark a more activated subpopulation of CD4 T cells. This, rather than upregulation upon viral integration, could explain the increased frequency of CD32^high^ cells observed during PHI (Figure 5A). Activated CD4 T cells are preferentially infected with HIV but rapidly die; the HIV reservoir is thought to form from cells that are infected while activated and then transition to a resting state (35, 38, 39), or while undergoing this transition (40). It is possible that with high cellular activation and high co-receptor expression, CD32^high^ cells might be more susceptible to infection with HIV. Whether these cells are able to persist and contribute to long-term latency remains unclear.

### HIV DNA in CD32-Expressing CD3^+^CD4^+^ Cell Subsets

In initial experiments using negative selection beads, sorting CD4 T cells into CD32^+^ and CD32^−^ fractions revealed an enrichment of HIV DNA in CD32^+^ cells from individuals who commenced ART during PHI (clinical characteristics in Table S1 in Supplementary Material; sort strategy in Figure S7A in Supplementary Material) (41). As well as concerns detailed above over the impact of the negative selection beads, we also determined that for individuals with extremely low yields of sorted CD32^+^CD3^+^CD4^+^ cells, low cell inputs into the qPCR resulted in over-estimation of both albumin and HIV DNA (Figure S8 in Supplementary Material), which would need to be accounted for when comparing different cell populations. We, therefore, re-analyzed these data (Table S2 in Supplementary Material) accounting for assay cell input (which was lower for the CD32^+^ compared with the CD32^−^ populations) and the number of positive qPCR replicates, and found that HIV DNA could still be detected within CD32^+^CD4 T cells, with some evidence for enrichment. All qPCR assays performed on sorted DNA included control wells without DNA template, which were consistently negative. To show that low inputs of HIV DNA did not give false positive signals, we diluted sorted CD32^−^ HIV DNA such that each well contained <1 copy of HIV DNA and found no detectable signal in 21 wells (*n* = 3 individuals, 2 or 3 triplicate dilutions per individual; from experiments in Figure S8B in Supplementary Material).

**Figure 4:**
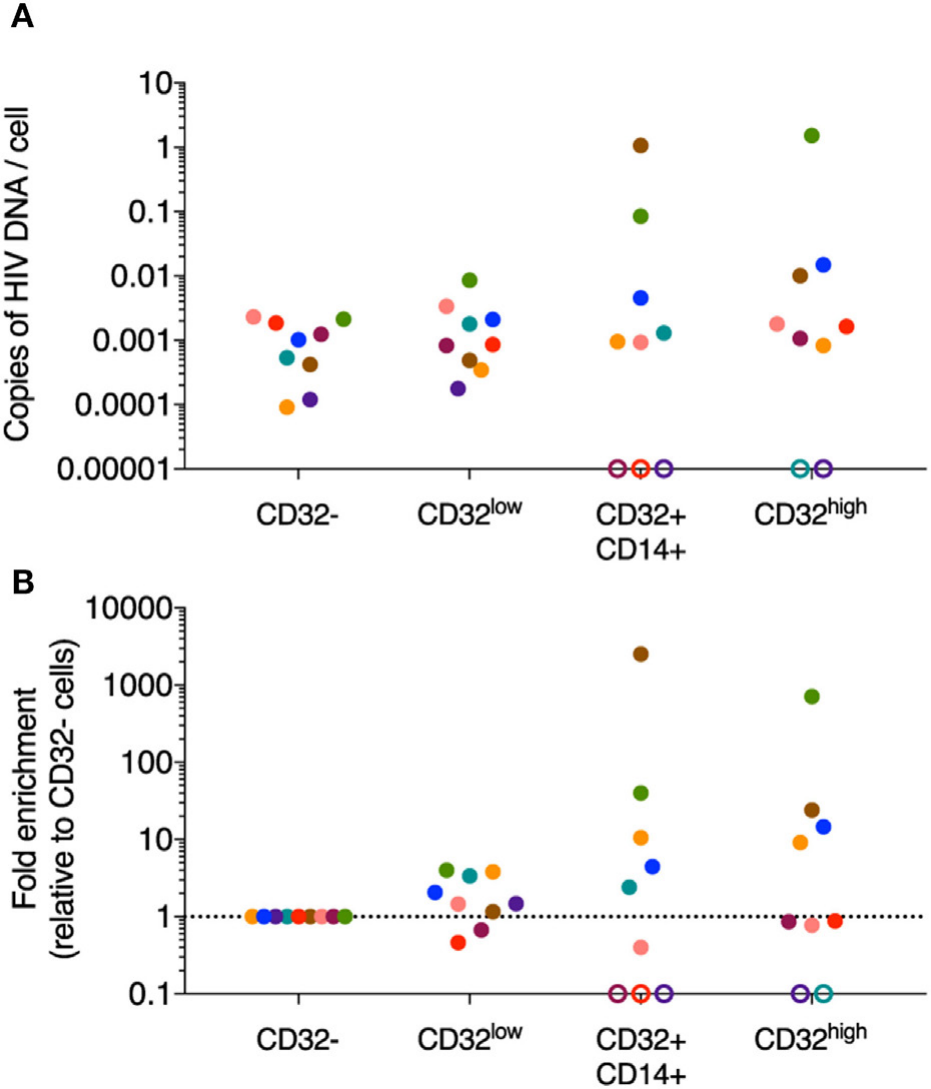
HIV DNA quantification in sorted CD32-expressing CD3^+^CD4^+^ T cell populations. HIV DNA was quantified in sorted CD32^−^, CD32^low^, CD32^+^CD14^+^, and CD32^high^ CD3^+^CD4^+^ T cell populations by qPCR (*n* = 9). **(A)** Shows the absolute quantification of these data and **(B)** shows the fold change relative to the CD32^−^ population. Copies of HIV-1 DNA were quantified and normalized to number of input cells (as determined by albumin PCR). PCR reactions for both albumin and total HIV were performed in triplicate for the CD32^−^ population and in duplicate for the remaining three populations. Data shown are averages of the replicates. Data points are colored by the individual from which the population was sorted. Where no signal was detectable for a given sorted population, this is shown as an open circle on the axis. For all of these undetectable populations, we could not determine if they were in fact negative for HIV DNA as the predicted level of infection (assuming equivalent level of infection to the CD32^−^ fraction) was below the level of detection of our assay based on the available input cell equivalents.

However, to negate the impact of negative selection beads on the CD32^+^ CD4 T cell population, all experiments were repeated sorting directly from bulk PBMCs. Cells from participants treated during PHI (Table S1 in Supplementary Material) were sorted directly into the four CD3^+^CD4^+^ populations (CD32^−^, CD32^low^, CD32^+^CD14^+^, and CD32^high^; Figure S7B in Supplementary Material) and HIV DNA quantified by qPCR. We were able to quantify HIV DNA in sorted CD32^−^, CD32^low^, CD32^+^CD14^+^, and CD32^high^ CD3^+^CD4^+^ T cells from most individuals (Figure 4A). As expected, numbers of sorted cells from these populations were higher than for previous experiments using CD4 negative selection kits alleviating concerns regarding low input bias in the qPCR (Table S3 in Supplementary Material). We observed some degree of HIV DNA enrichment within CD32^low^ cells from most individuals, although this was generally of low magnitude. CD32^+^CD14^+^ cells also contained detectable HIV DNA in most individuals. We also observed detectable HIV DNA in the CD32^high^ population from seven of nine individuals sorted, which was enriched in four of these. The degree of enrichment varied substantially between individuals (Figure 4B).

Despite the technical challenges detailed above, we were confident that HIV DNA was detectable in all three populations of CD32-expressing CD4 T cells and that some enrichment in CD32^high^ was observed in some individuals, although not in all. We have not, however, been able to replicate the magnitude of enrichment or the inter-individual consistency reported by Descours et al. (11), despite starting with a similar number of PBMCs. Importantly, our study population of participants with treated PHI also differs from this previous study which included greater heterogeneity in regards to timing of ART initiation.

### The Frequency of CD32-Expressing CD3^+^CD4^+^ Cells Is Not Related to the Overall Size of the HIV Reservoir

To further explore the proposed association with CD32 expression and the HIV reservoir, we studied additional individuals commencing ART during PHI from two clinical trials [SPARTAC (21) and HEATHER (7)]. Key demographic and clinical characteristics are shown in Table 1. The median time from estimated seroconversion to ART initiation was 74 days (interquartile range, IQR 36–114 days). Median baseline plasma viral load (pVL) was 4.94 log_10_(copies/mL) (IQR 4.09–6.52), and CD4 T cell count was 545 cells/μL (IQR 453–696). Clinical, immunological, and reservoir measures presented are at baseline (before the initiation of ART) and after 1 year of ART (median 48.1 weeks; IQR 47.9–52.9 weeks). Healthy controls [*n* = 10; 100% male; median age 34.5 (IQR 30.5–42.5) years] were included for comparison.

**Figure 5:**
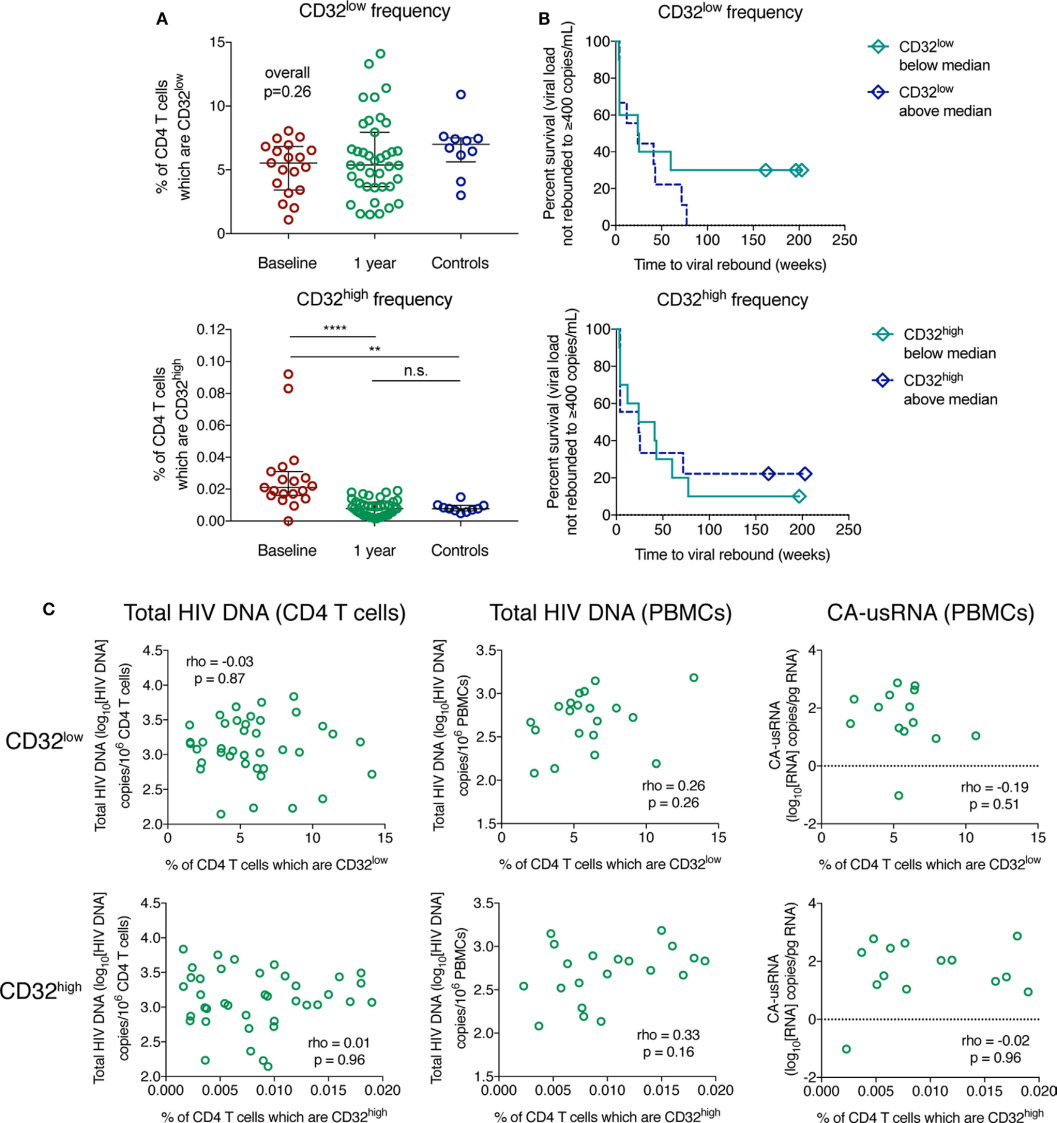
The frequency of CD32-expressing CD3^+^CD4^+^ cells does not correlate with the size of the HIV reservoir. The percentage of CD32^low^ (top panel) and CD32^high^ (bottom panel) CD3^+^CD4^+^ cell populations is shown in panel **(A)** for HIV^+^ individuals with primary HIV infection pre-therapy (baseline; *n* = 19) and 1 year following antiretroviral therapy initiation (*n* = 39), as well as for controls (*n* = 10); groups were compared using a Kruskal–Wallis test. Bars indicate the median with the interquartile range. Panel **(B)** shows survival curves (Kaplan–Meier) of time to viral rebound >400 copies/mL based on the frequency of CD32^low^ (top panel) and CD32^high^ (bottom panel) CD3^+^CD4^+^ cells stratified at the median (*n* = 19). The relationship between measures of the HIV reservoir and percentage of CD32^low^ (top panels) and CD32^high^ (bottom panels) CD3^+^CD4^+^ cells in treated HIV^+^ individuals, shown in panel **(C)**, was assessed using Spearman’s rank correlation. Left column shows total HIV DNA measured in isolated CD4 T cells (*n* = 39), the center column is total HIV DNA measured in peripheral blood mononuclear cells (PBMCs) (*n* = 20), and the right column shows cell-associated unspliced HIV RNA (*n* = 14); ****p < 0.0001, **p = 0.001–0.01, n.s. (not significant) indicates *p* ≥ 0.05.

For participants on ART, the median percentage of CD32^low^ cells was 5.38 (range 1.51–14.1%), and the rarer CD32^high^ population was 0.008% (range 0.002–0.02%). The frequency of CD32^low^ cells did not differ from pre-therapy levels (median 5.55%, range 1.09–8.05%) or healthy controls (median 7.00%, range 3.00–10.9%; Figure 5A; *p* = 0.26). Although unstimulated CD4 T cells are not generally expected to express CD32 (10), we are not the first to observe low-level expression of CD32 on CD4 T cells from healthy controls (42). The frequency of CD32^high^ cells was elevated during PHI relative to healthy controls (median 0.02%, range 0.01–0.09% versus median 0.007%, range 0.005–0.015%; *p* = 0.0004) but had decreased to similar levels (*p* < 0.0001) following 1 year of ART (Figure 5A).

Whether the percentage of CD32-expressing CD3^+^CD4^+^ cells predicted time to pVL rebound after ART TI was assessed in a subset of individuals after 48 weeks of suppressive ART (*n* = 19). In Cox models, we found no evidence to support such an association for either CD32^low^ or CD32^high^ cells (Figure 5B, Table S4 in Supplementary Material). Three individuals remained virologically suppressed at the end of follow-up after TI (median 197 weeks), all of whom expressed levels of CD32^low^ below the median (Figure 5B). We did not observe any correlation between the percentage of CD32^low^ or CD32^high^ cells and the overall size of the HIV reservoir on ART as measured by several assays: total HIV DNA in negatively selected CD4 T cells, total HIV DNA in PBMCs, and the level of cell-associated unspliced RNA (CA-usRNA) in PBMCs (Figure 5C; Figure S9 in Supplementary Material). We also measured HIV DNA in CD4 T cells before ART initiation and again observed no correlation between this and frequency of CD32-expressing populations (Figure S10 in Supplementary Material). In summary, we found no evidence that CD32 expression on CD4 T cells is a surrogate of the total reservoir size as measured by HIV DNA, CA-usRNA or time to viral rebound. This is likely to be not just because CD32^high^ cells are rare but that, even if enriched, a large part of the HIV reservoir is contained in other cell types.

## Conclusion

We have identified three distinct populations of CD32-expressing CD3^+^CD4^+^ T cells that differ in regards to their expression of lineage markers, ICRs, HLA-DR, and their memory phenotype. Our findings have implications for others studying these cells—particularly our finding that negative bead isolation of CD4 T cells will deplete a major fraction of CD32-expressing cells due to their expression of non-T cell markers. We have also shown that some CD32-expressing CD3^+^CD4^+^ cells also express CD14 and have a phenotype that suggests that these cells are monocytes. Efforts to ensure that these are excluded from analyses (which can be achieved with stringent gating) will be necessary in future studies.

The CD32^high^ population is of particular interest because of the previously reported high level of HIV DNA enrichment. The surface co-expression of both B and T cell markers is unusual, but has been previously reported (26–31), and further study into the biology of this population is warranted. In the absence of this functional characterization we are cautious in drawing any conclusions about the origin of these cells. Possibilities include that this phenotype is the result of a functional interaction between B cells and CD4 T cells, or represents an unusual lymphocyte with the capability to express markers of both lineages. With high levels of activation and HIV co-receptor expression it is possible that this population may be preferentially infected with HIV, although due to the rarity of this population we have not been able to directly test this.

We were able to identify HIV DNA in CD32^−^, CD32^low^, CD32^+^CD14^+^, and CD32^high^ cells. Although cautious about the conclusions that can be drawn from our data, we found evidence for some degree of enrichment of HIV DNA in CD32-expressing populations in some individuals, although we were not able to replicate the magnitude of enrichment previously reported. One possible explanation is that unlike Descours et al. (11), we studied only individuals who commenced ART during PHI, when the reservoir size is smaller (18, 19, 43, 44), and it is possible that the distribution and dynamics of reservoir formation and decay may also differ. We also highlight some of the technical challenges in quantifying exact levels of HIV DNA in rare cell populations which will need to be considered in future studies of the reservoir. Even if CD32-expressing CD4 T cells are enriched for HIV DNA in a given individual, it is likely that a large part of the reservoir is contained outside of this population. Supporting this, we found no evidence that CD32 expression on CD4 T cells is a surrogate of the total reservoir size as measured by HIV DNA, CA-usRNA or time to viral rebound. Overall, our data suggest that CD32 is not a specific marker of the reservoir but might identify a population of HIV enriched cells in a subset of patients.

## Data Availability Statement

The raw data supporting the conclusions of this manuscript will be made available by the authors on appropriate application. Data are from the SPARTAC and HEATHER studies from which unrestricted release of data for public deposition would breach compliance with the protocol approved by the research ethics boards. For SPARTAC, all trial data can be accessed by contacting hanna.box@imperial.ac.uk. For HEATHER, data can be accessed from the Collaborative HIV Eradication of Reservoirs: UK BRC steering committee by contacting john.frater@ndm.ox.ac.uk.

## Ethics Statement

Recruitment for and studies within the HEATHER cohort were approved by the West Midlands—South Birmingham Research Ethics Committee (reference 14/WM/1104). Tonsil tissue was obtained from one HIV^+^ individual undergoing routine tonsillectomy, 2 months after acquiring HIV, through the Imperial College Communicable Disease Group Biobank. The SPARTAC trial was approved by the following authorities: the Medicines and Healthcare products Regulatory Agency (UK), the Ministry of Health (Brazil), the Irish Medicines Board (Ireland), the Medicines Control Council (South Africa), and the Uganda National Council for Science and Technology (Uganda). It was also approved by the following ethics committees in the participating countries: the Central London Research Ethics Committee (UK), Hospital Universitário Clementino Fraga Filho Ethics in Research Committee (Brazil), the Clinical Research and Ethics Committee of Hospital Clinic in the province of Barcelona (Spain) and the Adelaide and Meath Hospital Research Ethics Committee (Ireland), the University of Witwatersrand Human Research Ethics Committee, the University of Kwazulu-Natal Research Ethics Committee, and the University of Cape Town Research Ethics Committee (South Africa), Uganda Virus Research Institute Science and ethics committee (Uganda), the Prince Charles Hospital Human Research Ethics Committee and St Vincent’s Hospital Human Research Ethics Committee (Australia), and the National Institute for Infectious Diseases Lazzaro Spallanzani, Institute Hospital and the Medical Research Ethics Committee, and the ethical committee of the Central Foundation of San Raffaele, MonteTabor (Italy). PBMCs used for characterisation of CD32-expressing CD3^+^CD4^+^ cell populations (Figures 1, 2, and 3G,H; Figures S1 and S2 in Supplementary Material) used in this study were isolated from leukocyte cones obtained through anonymous donation to NHS Blood and Transplant (UK) after approval by NHS Blood and Transplant and the National Research Ethics Service Oxfordshire Research Ethics Committee. Recruitment of healthy individuals (used as comparison group to primary HIV infection cohort and for fresh blood phenotyping; Figure 5A; Figures S3, S4E–G, and S5B in Supplementary Material) was approved by the Sheffield Research Ethics Committee (reference 16/YH/0247). All participants have given written informed consent for their participation in these studies.

## Author Contributions

The experiments were conceived and designed by GM, MP, JT, CW, PKl, and JFr. Experiments were performed by GM, MP, JT, CP, MG, HB, and NO and data analyzed by GM, MP, JT, KP, and JFr. Design and recruitment of the trials was performed by JL, NN, JFo, SF, GR, PKa, and JFr, with trial management performed by JM. The paper was written by GM, MP, JT, and JFr with input from all authors.

## Conflict of Interest Statement

The authors declare that the research was conducted in the absence of any commercial or financial relationships that could be construed as a potential conflict of interest.
