# Supplementary material for: CD32-Expressing CD4 T Cells Are Phenotypically Diverse and Can Contain Proviral HIV DNA
Source: Front Immunol. 2018 May 4;9:928. doi: 10.3389/fimmu.2018.00928 (PMC5946760; doi:10.3389/fimmu.2018.00928)
Supplement: Supplementary file 1 [file data_sheet_1.docx]

**Supplementary Table 1 - Demographic and clinical characteristics of participants included in additional experiments**

| **Experiments included in** | **Quantification of HIV DNA in sorted CD32+ and CD32- populations*** | **Quantification of HIV DNA in sorted CD32^low^, CD32+CD14+ and CD32^high^ populations** | **Measurement of HLA-DR expression** |
| --- | --- | --- | --- |
| Panels/references | Supplementary Table 2 | Figure 4, Supplementary Table 3 | Figure 3F, Supplementary Figure 5D |
| Sex |  |  |  |
| - Male | 6 (100%) | 9 (100%) | 19 (100%) |
| Age | 31 (26.5 – 38.5) | 30 (25 – 41) | 34 (27 – 41) |
| Country |  |  |  |
| - United Kingdom | 6 (100%) | 9 (100%) | 19 (100%) |
| Days between estimated date of seroconversion and ART initiation | 44.8 (17.3 – 62.0) | 38 (25.3 – 53.0) | 61 (22.5 – 133) |
| Time of sampling (weeks since ART initiation) | 67.4 (56.1 – 81.5) | 119 (75.6 – 299) | 51.4 (45 – 52.7) |
| Baseline CD4 T cell count (cells/μL) | 679 (574 – 882) | 587 (444 – 774) | 563 (476 – 724) |
| Baseline HIV RNA (log_10_ copies/mL) | 5.19 (4.38 – 5.56) | 6.67 (4.80 – 6.85) | 4.82 (4.28 – 5.86) |

Demographic and clinical characteristics of participants included in analysis of HIV DNA in sorted CD32 expressing populations or the measurement of HLA-DR expression. Values given represent n (%) for categorical variables and median (interquartile range) for continuous variables. * shows data for individuals whose PBMCs were sorted only (excludes tonsillar tissue). Tonsillar tissue was taken from an individual who had commenced antiretroviral therapy during primary HIV infection; of note, this individual was not virologically suppressed at the time of tonsillectomy (plasma VL 267 copies/ml).

**Supplementary Table 2. HIV DNA qPCR from sorted CD32- and CD32+ CD4+CD3+ populations following use of negative selection kits to isolate CD4 T cells.**

| **Participant ID** | **Frequency of HIV DNA+ve cells amongst CD32- population** | **Input number of CD32+ cells per well** | **Number of positive CD32+ wells** |
| --- | --- | --- | --- |
| ST417* | 1 in 85 | 35.2 | 0 of 1 |
| ST392 | 1 in 640 | 50 | 1 of 2 |
| ST364 | 1 in 591 | 53 | 1 of 2 |
| ST008 | 1 in 769 | 68 | 0 of 2 |
| SM107 | 1 in 6505 | 90 | 1 of 2 |
| ST420 | 1 in 252 | 174 | 2 of 2 |
| Tonsil | 1 in 165 | 1137 | 1 of 1 |

HIV DNA qPCR was performed on sorted CD32+ and CD32- populations following use of negative selection kits to isolate CD4 T cells. The number of cell equivalents of DNA in each reaction was calculated by albumin qPCR performed in duplicate (* indicates that only a single replicate was available for this sample). For each individual, the proportion of infected cells in the CD32- population is expressed as an infected cell frequency. Due to low input into the HIV DNA qPCR reactions for sorted CD32+ populations, the infected cell frequency was not able to be accurately calculated. Instead, the input number of CD32+ cell equivalents of DNA (calculated by albumin qpCR) for the HIV qPCR reaction in each well is shown, alongside the number of wells that were positive.

**Supplementary Table 3 - qPCR detection of HIV DNA for CD3+CD4+ T cell populations sorted based on CD32 expression directly from PBMCs**

| **Participant ID** | **Fold enrichment  (relative to CD32- population)** | | | |  | **Input number of cells per well** | | | |  | **Number of wells positive for HIV DNA** | | | |
| --- | --- | --- | --- | --- | --- | --- | --- | --- | --- | --- | --- | --- | --- | --- |
|  | CD32- | CD32^low^ | CD32+ CD14+ | CD32^high^ |  | CD32- | CD32^low^ | CD32+ CD14+ | CD32^high^ |  | CD32- | CD32^low^ | CD32+ CD14+ | CD32^high^ |
| SM005 | 1 | 3.82 | 10.51 | 9.13 |  | 25000 | 9260 | 1280 | 842 |  | 2 of 3 | 2 of 2 | 2 of 2 | 1 of 2 |
| ST344 | 1 | 1.16 | 2520.00 | 24.00 |  | 7890 | 1340 | 573 | 209 |  | 3 of 3 | 1 of 2 | 2 of 2 | 1 of 2 |
| SM065 | 1 | 3.35 | 2.41 | - |  | 25000 | 14000 | 2040 | 1330 |  | 3 of 3 | 2 of 2 | 2 of 2 | 0 of 2 |
| SM119 | 1 | 1.46 | 0.401 | 0.773 |  | 25000 | 8330 | 2420 | 7390 |  | 3 of 3 | 2 of 2 | 2 of 2 | 2 of 2 |
| ST028 | 1 | 1.48 | - | - |  | 25000 | 25300 | 3110 | 4230 |  | 2 of 3 | 2 of 2 | 0 of 2 | 0 of 2 |
| ST006 | 1 | 0.67 | - | 0.86 |  | 25000 | 7250 | 884 | 1230 |  | 3 of 3 | 2 of 2 | 0 of 2 | 1 of 2 |
| ST414 | 1 | 2.06 | 4.45 | 14.6 |  | 10300 | 2190 | 320 | 243 |  | 3 of 3 | 2 of 2 | 1 of 2 | 1 of 2 |
| ST420 | 1 | 4 | 40 | 712 |  | 25000 | 304 | 97 | 136 |  | 3 of 3 | 2 of 2 | 2 of 2 | 2 of 2 |
| SM146 | 1 | 0.46 | - | 0.88 |  | 25000 | 40000 | 1240 | 973 |  | 3 of 3 | 2 of 2 | 0 of 2 | 1 of 2 |

qPCR for HIV DNA was performed on sorted CD3+CD4+ T cell populations. The number of cell equivalents of DNA in each reaction was calculated by albumin qPCR. For each individual and cell population the table shows a) the fold enrichment of HIV DNA relative to CD32- population b) the input number of cell equivalents of DNA (calculated by albumin qpCR) for the HIV qPCR reaction in each well is shown and c) the number of wells that were positive. Where the level of HIV DNA was not detectable, fold enrichment could not be calculated and is shown as - .

**Supplementary Table 4 – Cox proportional hazard models of time to viral rebound**

| **Variable** | **Time measured** | **Hazard ratio (95% confidence interval)** | **p-value** |
| --- | --- | --- | --- |
| Percentage of CD4 T cells which are CD32^low^ | 48 weeks (treatment interruption) | 1.0 (0.91 – 1.2) | 0.53 |
| Percentage of CD4 T cells which are CD32^high^ | 48 weeks (treatment interruption) | 6 x 10^-19^ (7 x 10^-72^ -5.2 x 10^34^) | 0.50 |
| CD4 T cell count (100 cells/μL) | Baseline | 0.90 (0.64 – 1.3) | 0.56 |
| Viral load (log_10_[copies/mL]) | Baseline | 1.7 (0.99 - 2.9) | 0.053 |
| Total HIV DNA (log_10_[copies/10^6^ CD4 T cells]) | 48 weeks (treatment interruption) | 1.2 (0.29 – 4.8) | 0.82 |

Univariable Cox proportional hazard models of variables listed and time to viral rebound >400 copies/mL. n=19 for all analyses. Hazard ratios correspond to a change of 100 cells/μL for CD4 T cell count or a log for viral load and total HIV DNA.

**Supplementary Figure 1 – Expression of non-T cell markers on populations of CD32 expressing CD3+CD4+ T cells**

**
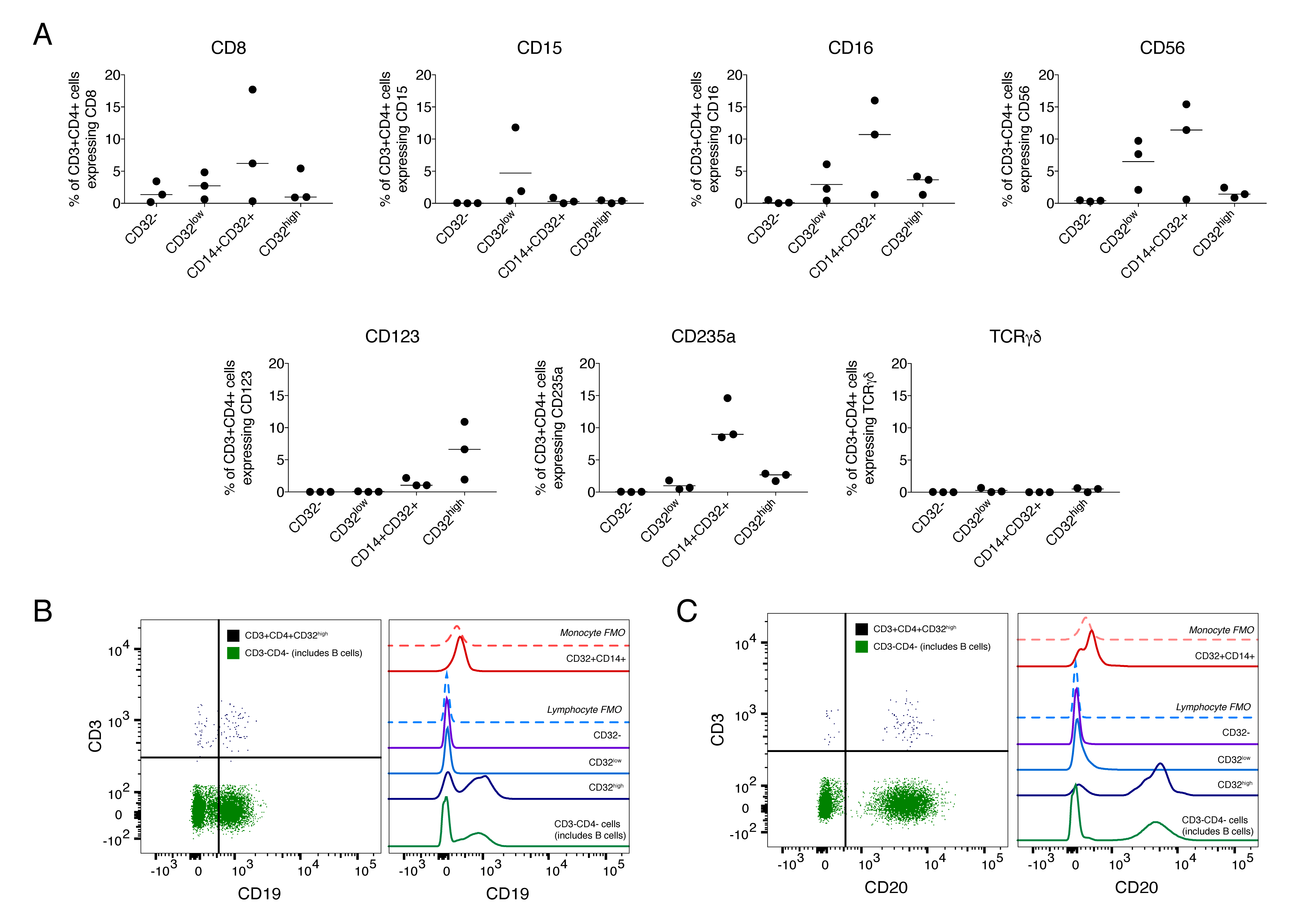
**

(A) Percentage expression of CD8, CD15, CD16, CD56, CD123, CD235a and TCRϒδ on CD32 expressing CD3+CD4+ subpopulations as summarised in Figure 1D. Expression was measured by flow cytometry on cells from three individual donors and bar is shown at the mean. (B) Overlaid is representative staining of CD3 and CD19 on CD3+CD4+CD32^high^ cells (purple) and CD3-CD4- cells (green, which includes the B cells as a positive control). The histogram shows CD19 staining on all CD32 expressing CD3+CD4+ T cell populations. Fluorescence minus one (FMO) controls are shown as dashed lines and individual populations as solid lines. (C) Overlaid is representative staining of CD3 and CD20 on CD3+CD4+CD32^high^ cells (purple) and CD3-CD4- cells (green, which includes the B cells as a positive control). The histogram shows CD20 staining on all CD32 expressing CD3+CD4+ T cell populations. Fluorescence minus one (FMO) controls are shown as dashed lines and individual populations as solid lines.

**Supplementary Figure 2 – CD14+CD32+ CD3+CD4+ cells have forward and side scatter characteristics which fall outside a conservative lymphocyte gate**

**
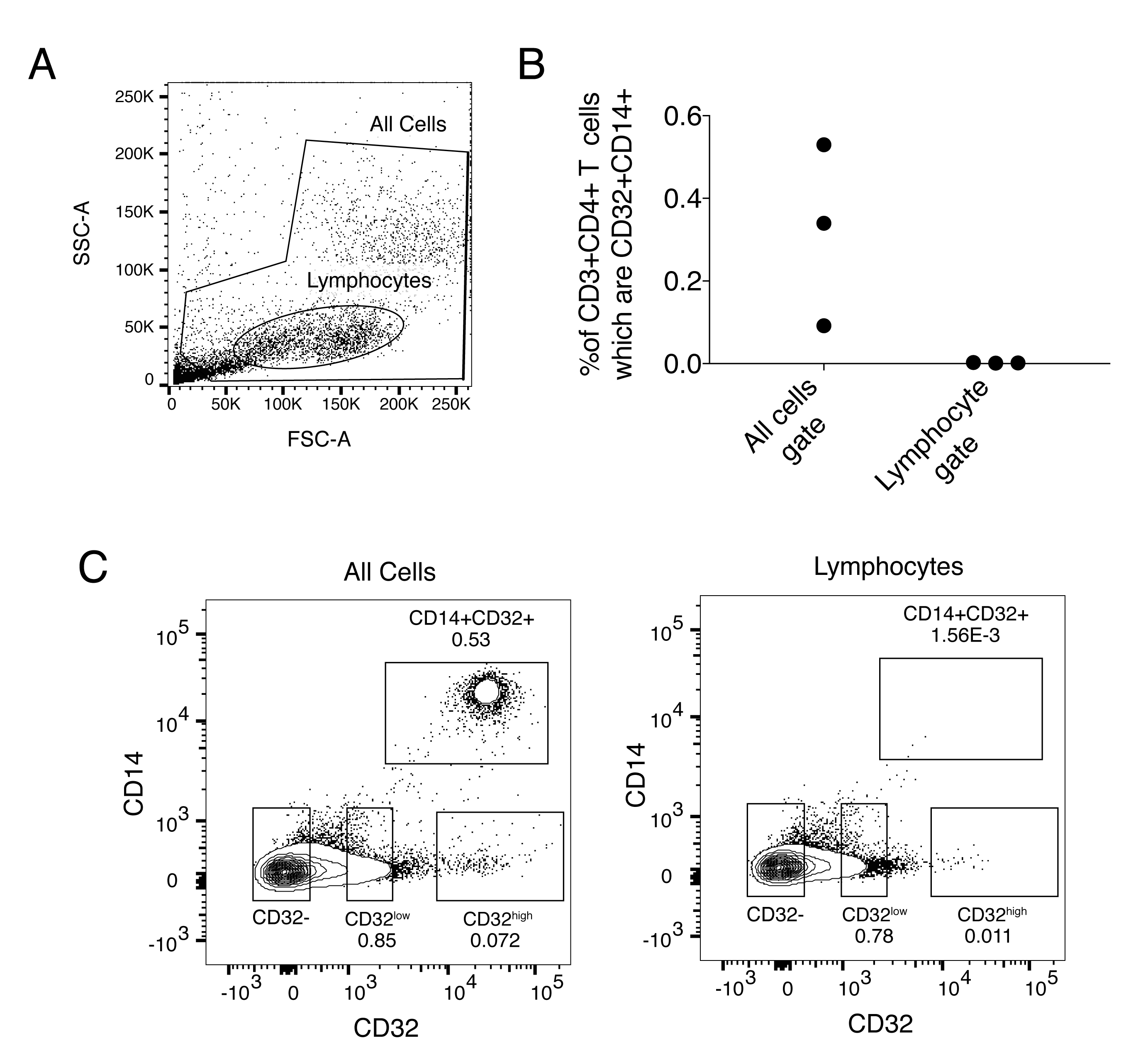
**

CD32 expressing populations of CD3+CD4+ T cells are shown with different FSC-A and SSC-A gates. (A) Two different gates are shown based on FSC-A and SSC-A: Lymphocytes and All Cells. The All Cells gates includes the lymphocytes but also contains the monocyte population. (B) The percent of CD3+CD4+ T cells which are CD32+CD14+ when the same samples are gated these two different ways. Expression is shown from three individual donors. (C) Representative gating from one individual shown in B. Abbreviations: FSC-A (forward scatter, area), SSC-A (side scatter, area).

**Supplementary Figure 3 – Doublet exclusion strategy as used in phenotyping experiments**

**
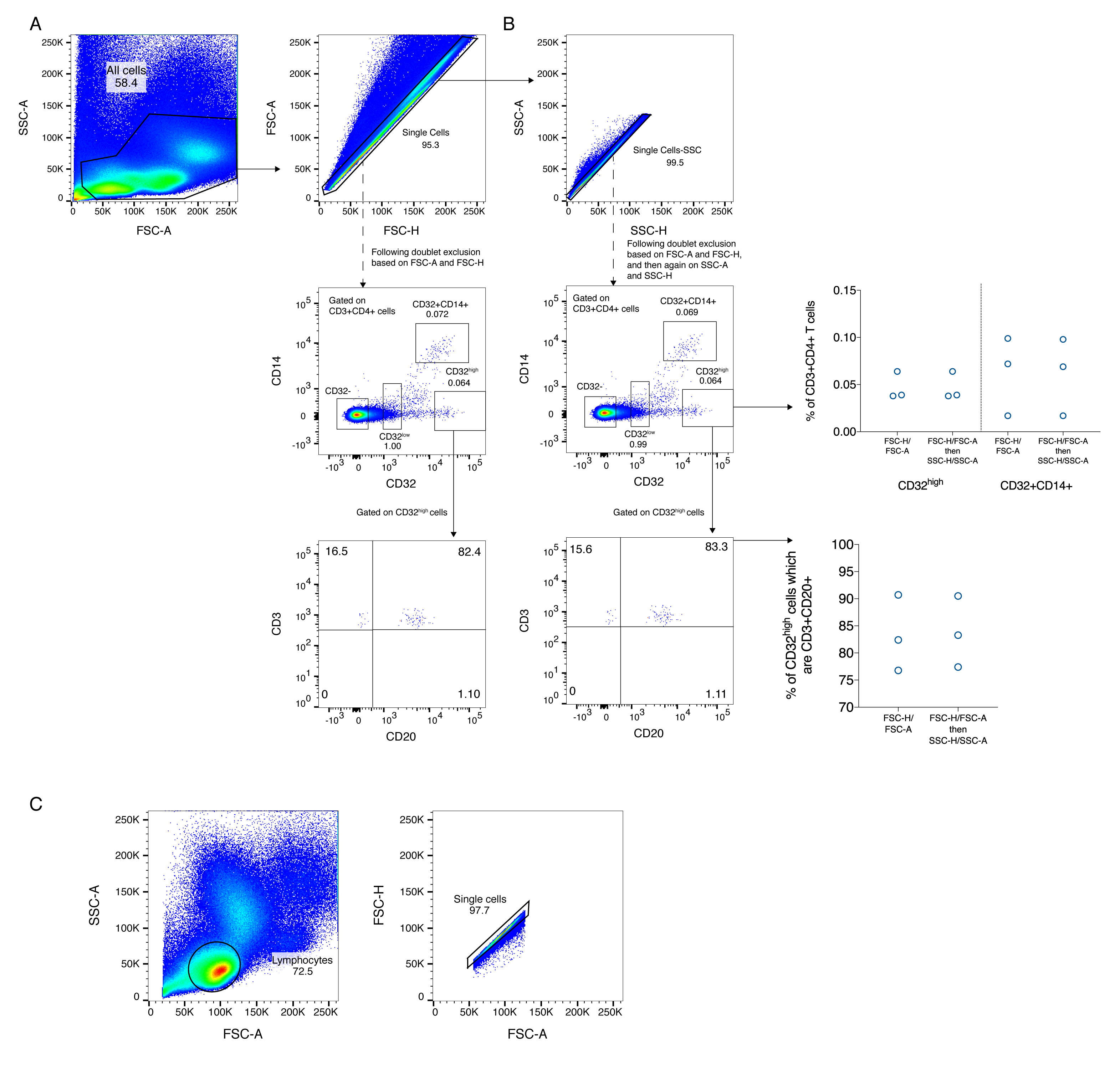
**

Representative doublet exclusion based on FSC-H and FSC-A for experiments presented in Figures 1, 2 and Supplementary Figures 1, 2 and 4. Below these gates are the phenotype of CD3+CD4+ T cells with regard to CD14 and CD32 expression, and CD20 expression. Shown in parallel in Panel (B) are the same downstream gates if a second doublet exclusion step based on SSC-H and SSC-A is performed. The right hand side of this panel shows a quantification the frequency of these populations for n=3 donors for both the single and sequential doublet exclusion steps. Panel (C) shows a representative plot of doublet exclusion based on FSC-H and FSC-A for experiments presented in Figures 3 and 5, and Supplementary Figures 5 and 6.

**Supplementary Figure 4 – CD32 expressing CD4 T cells from whole, fresh blood have a similar phenotype to those from cryopreserved PBMCs**

**
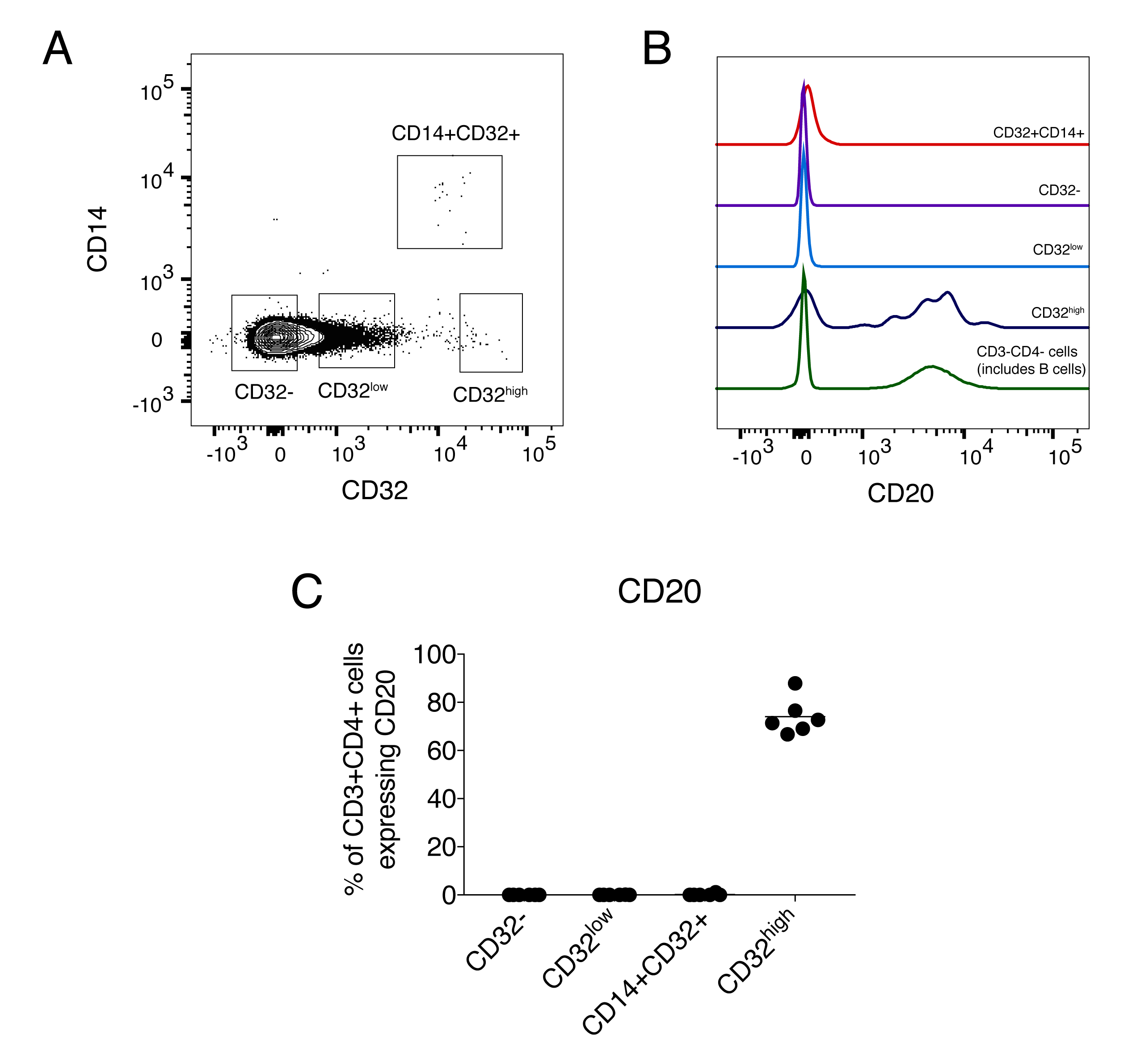
**

Phenotyping of CD32 expressing CD3+CD4+ T cells from whole, fresh blood (no cryopreservation or density gradient isolation of lymphocytes). (A) Gating of three CD32 expressing populations based on differing levels of expression and CD14: CD32^low^, CD32+CD14+ and CD32^high^. Plot shown from one representative donor. (B) Histogram showing CD20 staining on all CD32 expressing CD3+CD4+ T cell populations. Plot shown from one representative donor. (C) Percentage expression of CD20 on CD3+CD4+ subpopulations from 6 donors. Bar is shown at the mean.

**Supplementary Figure 5 – Immune checkpoint receptor expression on CD32-, CD32^low^ and CD32^high^ CD3+CD4+ T cell populations prior to the initiation of antiretroviral therapy and in healthy controls**

**
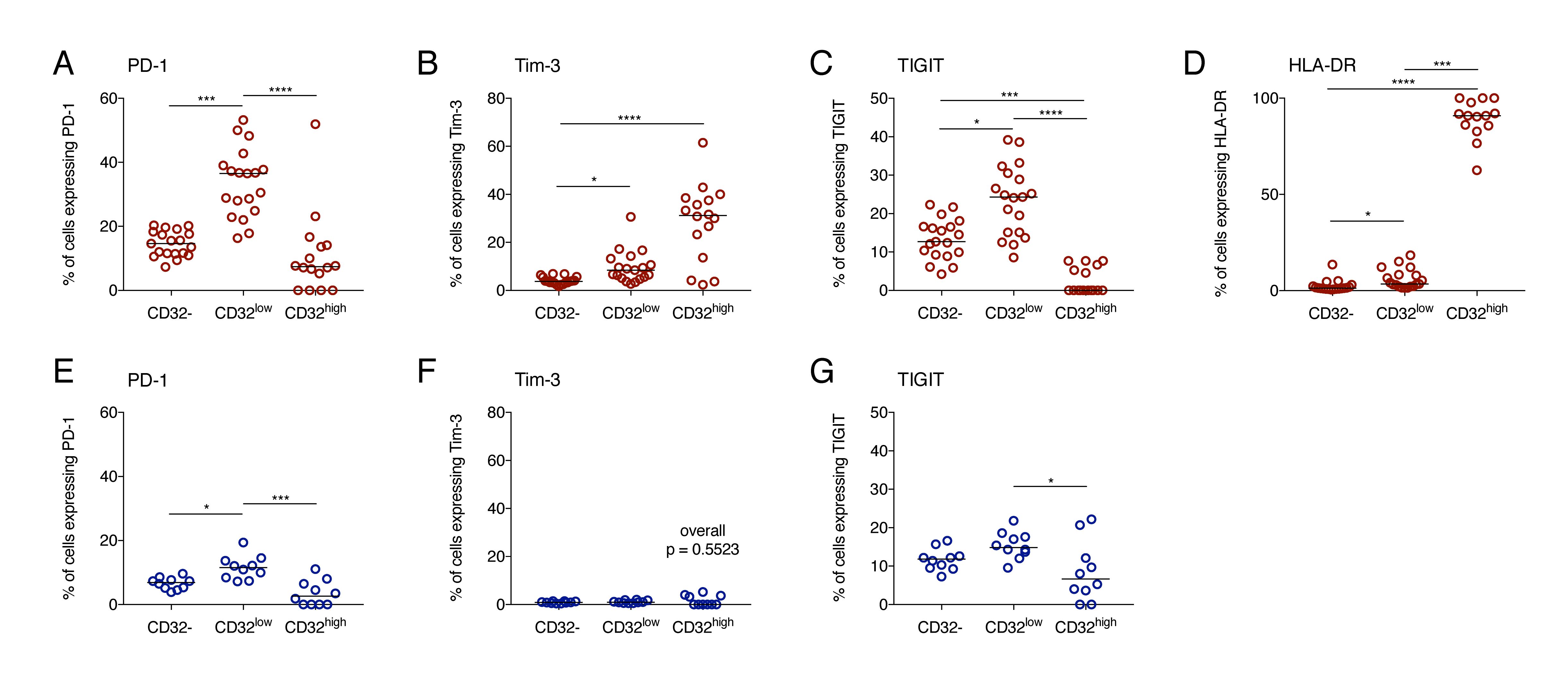
**

CD32-, CD32^low^ and CD32^high^ CD3+CD4+ T cell populations compared with regards to their immune checkpoint receptor and HLA-DR expression. The expression of PD-1 (A and E), Tim-3 (B and F), TIGIT (C and G) and HLA-DR (D) was quantified by flow cytometry and is shown for individuals with primary HIV infection (PHI) prior to the initiation of antiretroviral therapy (A-D, n=19). In panels A-C, 3 samples were excluded from the analysis of the CD32^high^ population because there were 5 or fewer events; in panel D 5 samples were excluded from the analysis of the CD32^high^ population. E-G show these same comparisons for healthy controls (n=10). To allow for visual comparison of the overall levels of expression of these markers, the axis range used is the same for both groups. Throughout, a Kruskal-Wallis test was used to compare all three groups; pairwise comparisons were performed on all combinations of groups only if the overall test p-value was <0.05. **** indicates p<0.0001, *** indicates p = 0.0001-0.001, ** indicates p = 0.001-0.01, * indicates p = 0.01 – 0.05; for ease of interpretation if p≥0.05 for any comparison this is not shown on these plots.

**Supplementary Figure 6 – Memory differentiation of CD32-, CD32^low^ and CD32^high^ CD3+CD4+ T cell populations prior to the initiation of antiretroviral therapy and in healthy controls**


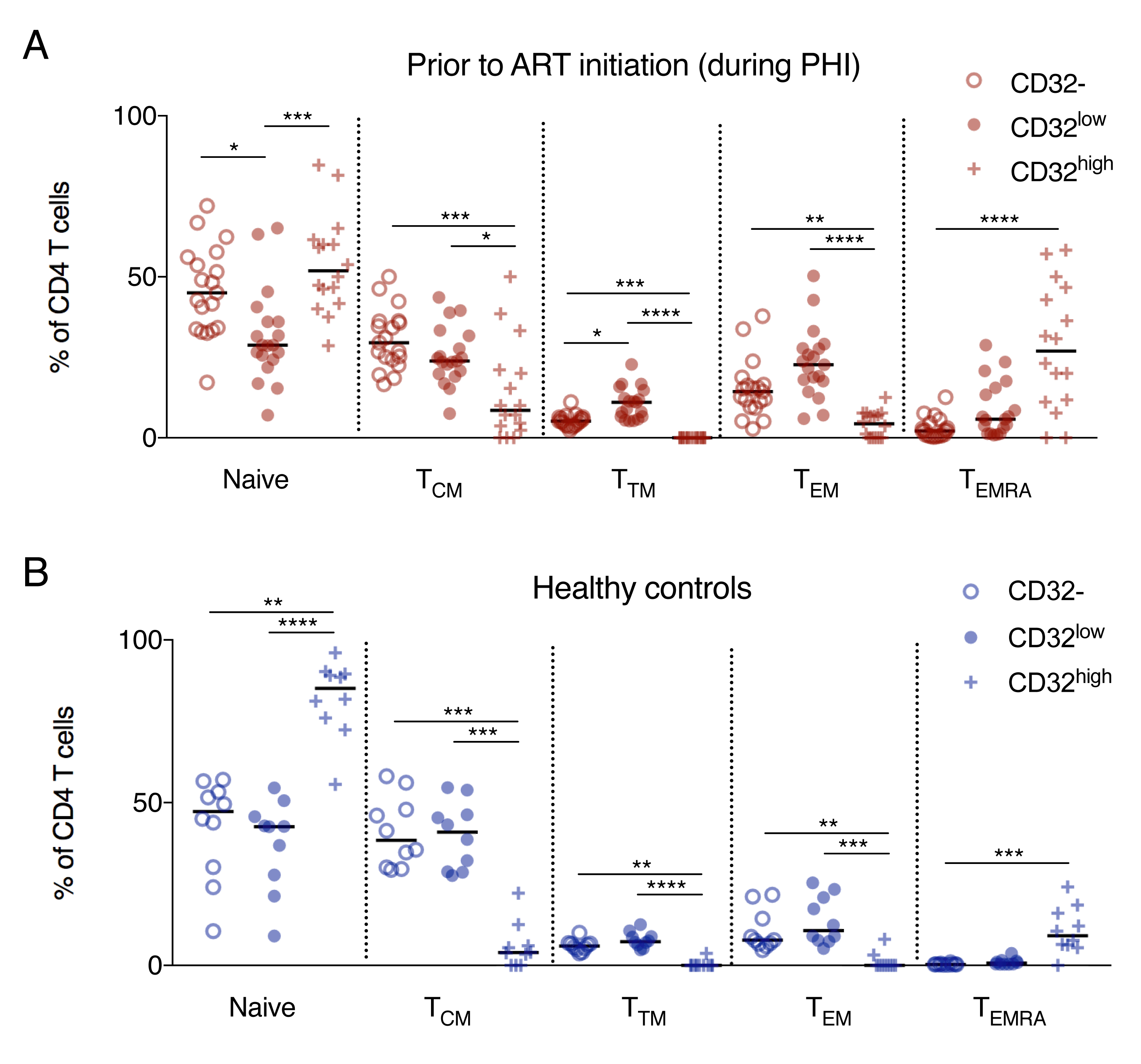


CD32-, CD32^low^ and CD32^high^ CD3+CD4+ T cell populations compared with regards to their memory phenotype. The percentage of each population comprised of naïve (CD45RA+CCR7+), central memory (T_CM_; CD45RA-CCR7+), transitional memory (T_TM_; CD45RA-CCR7-CD27+), effector memory (T_EM_; CD45RA-CCR7-CD27-) and T_EMRA_ cells (CD45RA+CCR7-) was quantified by flow cytometry and is shown for individuals with primary HIV infection (PHI) prior to the initiation of antiretroviral therapy in A (n=19). Three samples were excluded from the analysis of the CD32^high^ population because there were 5 or fewer events. (B) shows these same comparisons for healthy controls (n=10). Throughout, a Kruskal-Wallis test was used to compare all three groups; pairwise comparisons were performed on all combinations of groups only if the overall test p-value was <0.05. **** indicates p<0.0001, *** indicates p = 0.0001-0.001, ** indicates p = 0.001-0.01, * indicates p = 0.01 – 0.05; for ease of interpretation if p≥0.05 for any comparison this is not shown on these plots.

**Supplementary Figure 7 – representative gating for sorts of CD3+CD4+ T cells based on CD32 expression**

**
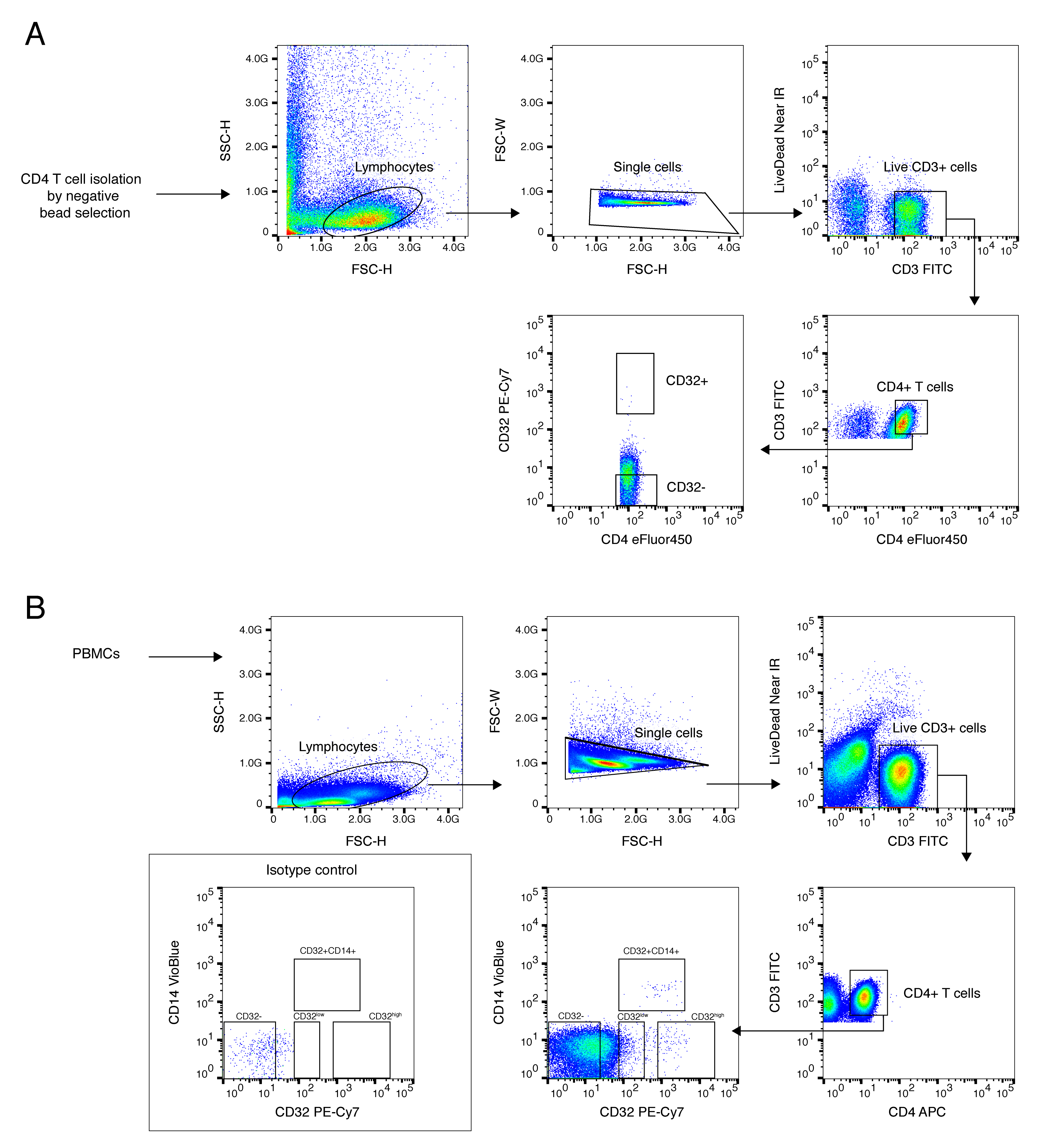
**

Representative gating used for sorts of CD32 expressing CD3+CD4+ populations for HIV DNA quantification. (A) Sort strategy used for initial sorts of CD32+ and CD32- populations on negatively selected CD4 T cells. (B) Sort strategy used for sorting of CD32-, CD32^low^, CD32+CD14+ and CD32^high^ populations. Both panels show images from a single participant.

**Supplementary Figure 8 – Low cell input results in overestimation of HIV and albumin DNA by qPCR**


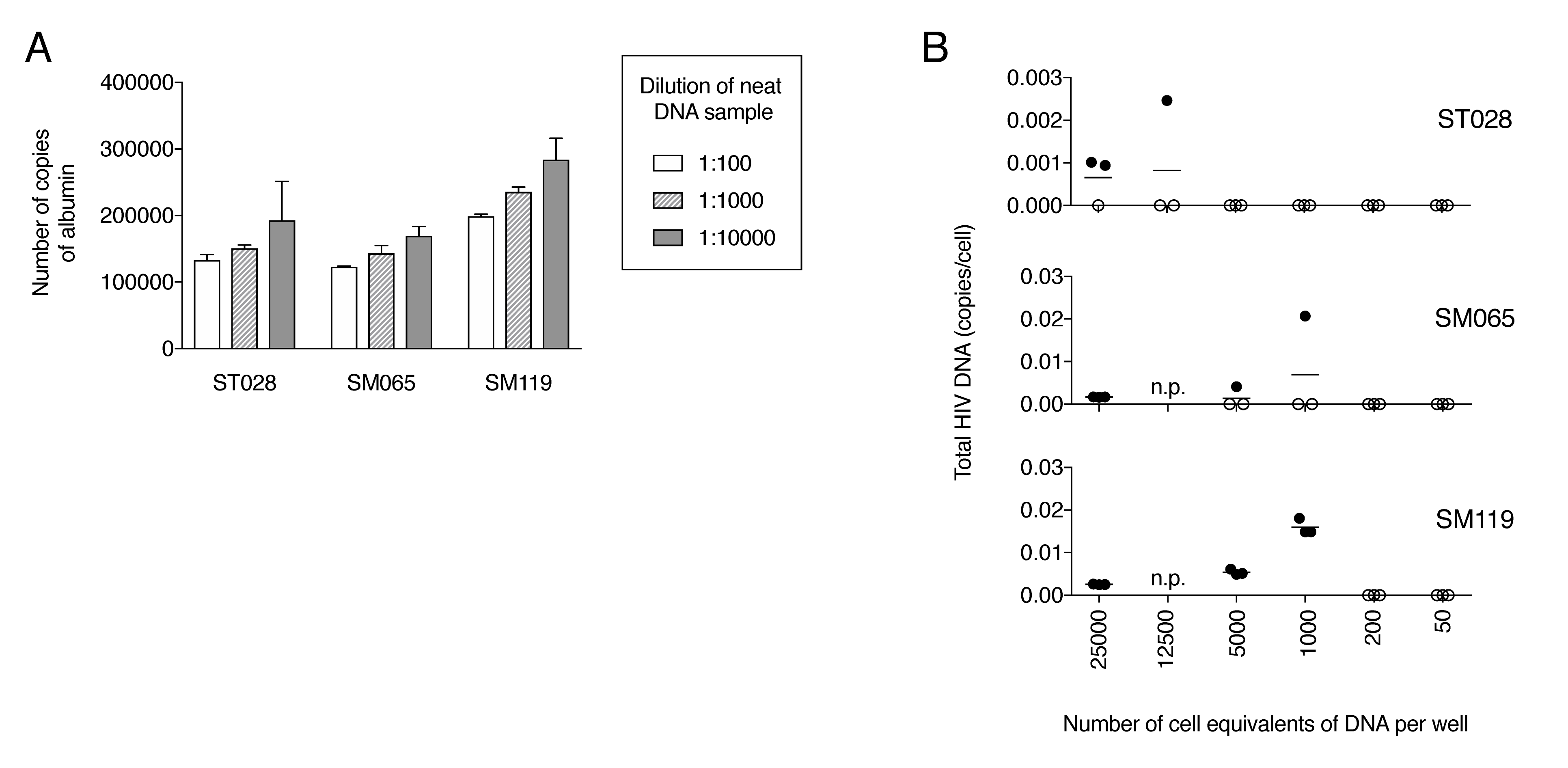


Quantification of albumin and HIV DNA in serially diluted samples of DNA extracted from sorted CD32- CD4 T cells. (A) Quantification of number of copies of albumin DNA (used as a measure of cell equivalents of DNA) using qPCR when neat sample (153000, 242000, and 137000 cells per well for ST028, SM065 and SM119 respectively) was diluted 1:100, 1:1000 or 1:10000. Data are shown for three individuals (labelled on x-axis) and each dilution was performed in triplicate. Values shown are mean of replicates and error bars indicate the standard deviation. (B) Quantification of number of copies of HIV DNA via qPCR with decreasing cell equivalents of DNA used as input, starting with our usual assay input of 25000 cell equivalents per well. Data are shown for three individuals. Dilutions were performed in triplicate and each data point indicates an individual replicate; open circles indicate that the well was negative; n.p. indicates that a given dilution was not performed for that sample. Bar shows the mean of replicates.

**Supplementary Figure 9 – Correlations between different measures of HIV reservoir size**


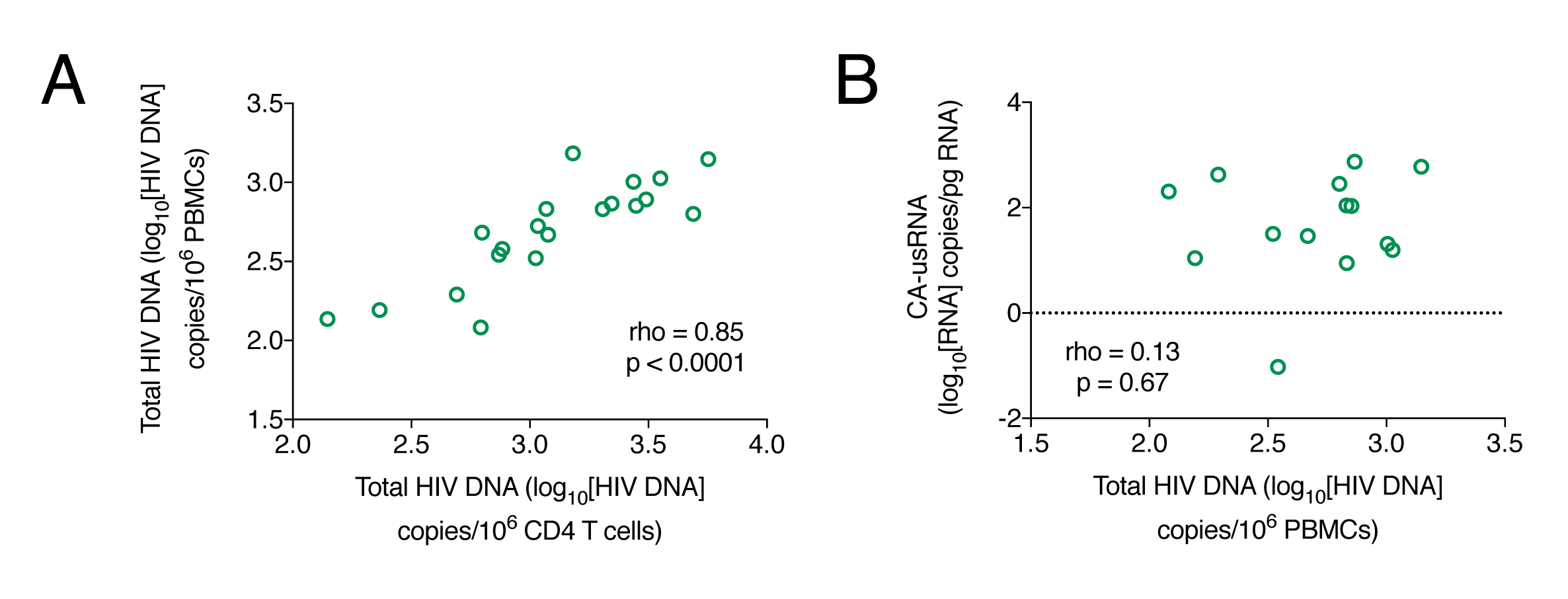


(A) Total HIV DNA measured in isolated CD4 T cells, and in PBMCs (n=20) at 1 year following antiretroviral therapy initiation. (B) Total HIV DNA and cell associated unspliced HIV RNA (CA-usRNA) measured in PBMCs (n=14) at 1 year following antiretroviral therapy initiation. CA-usRNA measures were unavailable for some individuals due to lack of sample availability (n=1) or no detectable PCR signal (n=5). For both A and B correlative analyses were performed using Spearman’s rank correlation.

**Supplementary Figure 10 – Correlations between proportions of CD32 expressing CD3+CD4+ T cells prior to the initiation of antiretroviral therapy**

**
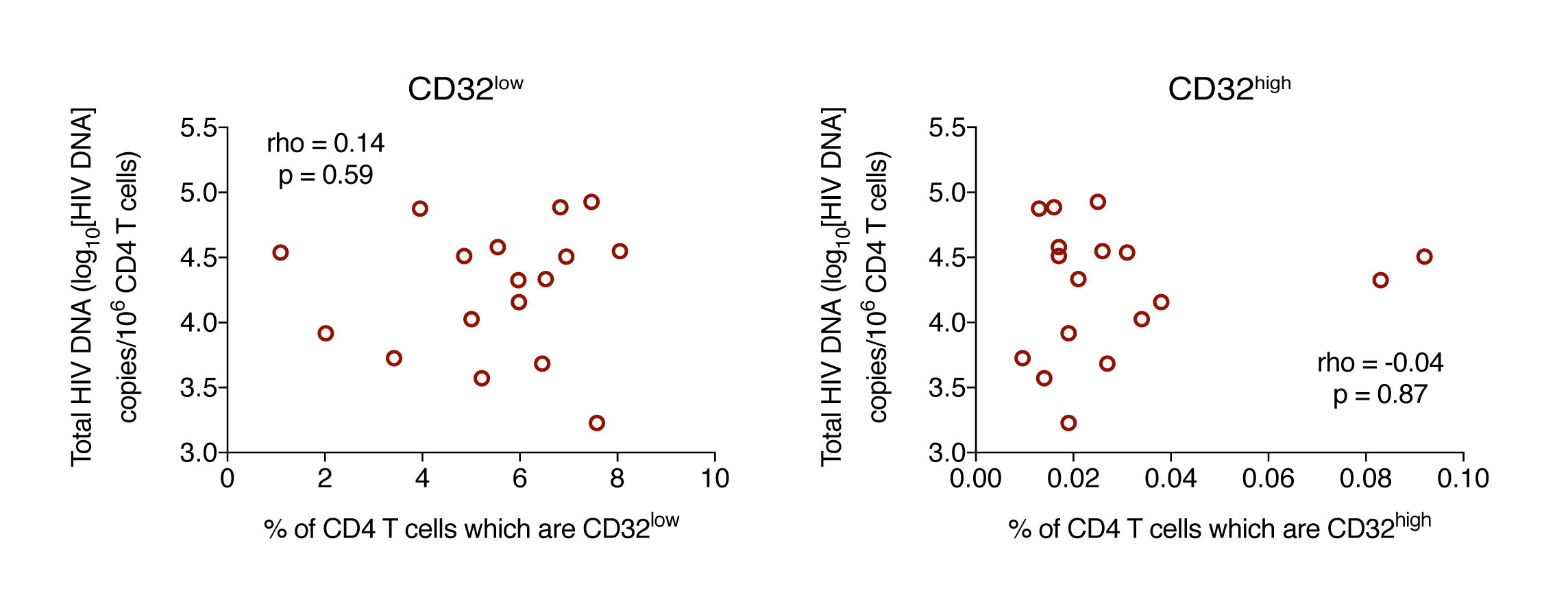
**

The proportion of CD32^low^ (left panel) and CD32^high^ (right panel) CD3+CD4+ T cells are shown against Total HIV DNA measured in CD4 T cells, both measured prior to the initiation of antiretroviral therapy. Correlative analyses were performed using Spearman’s rank correlation.
